# Assembly and activation of the Hippo signalome by FAT1 tumor suppressor

**DOI:** 10.1038/s41467-018-04590-1

**Published:** 2018-07-09

**Authors:** Daniel Martin, Maria S. Degese, Lynn Vitale-Cross, Ramiro Iglesias-Bartolome, Juan Luis Callejas Valera, Zhiyong Wang, Xiaodong Feng, Huwate Yeerna, Vachan Vadmal, Toshiro Moroishi, Rick F. Thorne, Moraima Zaida, Bradford Siegele, Sok C. Cheong, Alfredo A. Molinolo, Yardena Samuels, Pablo Tamayo, Kun Liang Guan, Scott M. Lippman, J. Guy Lyons, J. Silvio Gutkind

**Affiliations:** 1 Oral and Pharyngeal Cancer Branch, National Institutes of Health, Bethesda, MD 20892 USA; 2 Laboratory of Cellular and Molecular Biology, Center for Cancer Research, National Cancer Institute, National Institutes of Health, Bethesda, MD 20892 MD USA; 3 University of California San Diego, Moores Cancer Center, La Jolla, CA 92093 USA; 4 Department of Pharmacology, University of California, San Diego, CA 92093 USA; 5 School of Environmental and Life Sciences, University of Newcastle, and Hunter Cancer Research Alliance, Callaghan, NSW 2308 Australia; 6 Department of Pathology School of Medicine, University of Colorado, Colorado, CO 80045 USA; 7 Oral Cancer Research Team, Cancer Research, Selangor, Malaysia; 8 Department of Oro-maxillofacial Surgery and Medical Sciences, Faculty of Dentistry, University of Malaya, Kuala Lumpur, Malaysia; 9 Department of Molecular Cell Biology, The Weizmann Institute of Science, Rehovot, 76100 Israel; 10 Dermatology, Bosch Institute, University of Sydney, Camperdown, NSW 2050 Australia; 11 Cancer Services, Royal Prince Alfred Hospital, Camperdown, NSW 2050 Australia; 12 Centenary Institute, Camperdown, NSW 2050 Australia

## Abstract

Dysregulation of the Hippo signaling pathway and the consequent YAP1 activation is a frequent event in human malignancies, yet the underlying molecular mechanisms are still poorly understood. A pancancer analysis of core Hippo kinases and their candidate regulating molecules revealed few alterations in the canonical Hippo pathway, but very frequent genetic alterations in the FAT family of atypical cadherins. By focusing on head and neck squamous cell carcinoma (HNSCC), which displays frequent *FAT1* alterations (29.8%), we provide evidence that *FAT1* functional loss results in YAP1 activation. Mechanistically, we found that FAT1 assembles a multimeric Hippo signaling complex (signalome), resulting in activation of core Hippo kinases by TAOKs and consequent YAP1 inactivation. We also show that unrestrained YAP1 acts as an oncogenic driver in HNSCC, and that targeting YAP1 may represent an attractive precision therapeutic option for cancers harboring genomic alterations in the *FAT1* tumor suppressor genes.

## Abstract (web summary)

Dysregulation of the Hippo signaling is a frequent event in human malignancies, but the molecular mechanisms remain unclear. Here the authors show that in head and neck squamous carcinoma, FAT1 interacts with the Hippo signaling complex, resulting in the activation of core Hippo kinases and YAP1 inactivation.

## Introduction

Persistent activation of YAP1 and its paralog WWTR1 (also known as TAZ), is a hallmark of multiple human malignancies^1–3^. However, the molecular mechanisms driving YAP1 activation in cancer are still poorly defined. Genetic analysis in Drosophila revealed that the activity of Yorkie (Yki), the Drosophila YAP1 ortholog, is controlled by an intricate molecular network collectively known as the Hippo pathway^4^. Mammalian cells, however, appear to have evolved to fine tune the activity of YAP1 by multiple signals under physiological conditions, including growth promoting and inhibitory factors, matrix composition, cell–cell contact, cell density, mechanical perturbation, and metabolic conditions, to name but a few^5^. The highly conserved core Hippo kinase cascade is initiated by the activation of the mammalian Hippo orthologs, MST1 and MST2 (MST1/2), which are associated with the adaptor protein WW45/SAV1. MST1/2 phosphorylates and activates LATS1/2 kinases, referred to herein as LATS, in complex with MOB. In turn, active LATS phosphorylates and inhibits the mammalian transcription co-activator YAP1 and its related protein TAZ, which are degraded or excluded from the nucleus, thereby preventing their association with their target transcription factors, including TEAD family members^6^. In light of the crucial role of MST1/2 and LATS in YAP1 regulation, there are surprisingly few recurrent alterations in these core Hippo pathway components in cancer^1^. Indeed, there are only a few examples of known YAP1 regulating genes altered in cancer, which include LATS2 and an upstream Hippo pathway component, NF2, in malignant mesothelioma (35% and 50%, respectively)^7^, and inherited NF2 mutations and microdeletions in neurofibromatosis type 2^8^, overall accounting for a small fraction of human malignancies displaying YAP1 hyperactivity.

Here, we identify the alteration of FAT1 as a recurrent event in human cancer acting in coordination with other YAP1 activating mechanisms. We found that in normal conditions, FAT1 enables the assembly of a signaling complex including the canonical Hippo signaling components leading to phosphorylation and inactivation of YAP1. Gene deletions or truncating mutations of FAT1 result in impaired regulation of YAP1 activity. The high prevalence of these alterations underscore the crucial role of this oncogenic mechanism in human malignancies. Finally, we show that targeting unrestrained YAP1 may represent an attractive precision therapeutic option for cancers harboring genomic alterations in the FAT1 tumor suppressor genes.

## Results

### Widespread alterations in *FAT1* in cancer

**Fig. 1:**
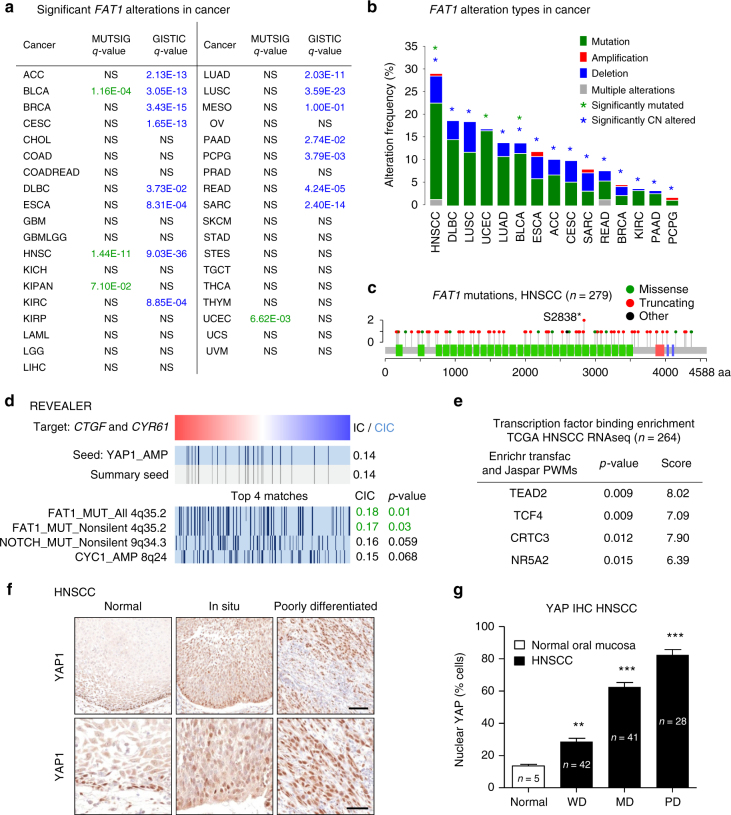
Frequent alterations of *FAT1* are linked to YAP1 overactivity in cancer. **a** Analysis of *FAT1* alterations in human malignancies. The significance of *FAT1* alterations in a panel of common human malignancies was analyzed by the MUTSIG and GISTIC methods. NS: not significant. See abbreviations and number of cases analyzed for each cancer type in Supplementary Fig. 1. **b** Graphical representation of cancer types in which *FAT1* is most frequently altered. **c** Analysis of FAT1 mutations in 279 fully characterized HNSCC samples from TCGA. **d** REVEALER analysis on the TCGA HNSCC RNASeq dataset (*n* = 504) was used to identify genomic abnormalities that negatively correlate with YAP1 amplification and increased expression of the YAP1 targets *CTGF* and *CYR61*. **e** Enrichment analysis of transcription factor binding sites on upregulated genes in HNSCC samples harboring *FAT1* and *FAT2* alterations. **f** YAP1 immunohistochemistry depicting the increase of expression levels and nuclear localization (activation) during HNSCC disease progression. Scale bar upper panels, 100 µm, lower panel, 50 µm. **g** YAP1 staining quantification. WD, well differentiated; MD, moderately differentiated; PD, poorly differentiated. Bars represent average plus standard error of the mean (SEM). **P < 0.01, ***P < 0.001 (One-way ANOVA)

As an approach to explore the molecular mechanisms resulting in tumor-associated YAP1 activation, we investigated the presence of genomic alterations in all human orthologs of Drosophila Hippo pathway components in a large panel of 38 distinct cancers sequenced by The Cancer Gene Atlas consortium (TCGA, 14729 neoplastic lesions, Supplementary Fig. 1a)^9^. Among these genes, a recently developed mutation significance method (MutSigCV), which provides a statistical metric to identify driver candidates in cancer with respect to the gene nucleotide length and the background mutation rate of each cancer analyzed^10^, recognized only *FAT1* to be significantly mutated when conducting a pancancer analysis (Supplementary Fig. 1a and b, and see below, Fig. 1a). Of interest, some members of the canonical Hippo pathway also achieved statistical significance when analyzing each cancer type individually (Supplementary Fig. 1c), suggesting their potential role in YAP activation in these specific cases. In addition to mutations, we also studied somatic copy number alterations predicted by the GISTIC2.0 method^11^. We found many known or candidate YAP1 and FAT regulators or associated transcription factors to be significantly amplified (*MST2*/*STK3*, *TEAD4*, *TAZ*/*WWTR1*, and *YAP1*) or deleted (*DCHS2*, *FAT1*, *FAT4*, *LATS1/2*, *TEAD1*, *TEAD2*, and *KIBRA*/*WWC1*) (Supplementary Fig. 1b). However, most of these copy number variations involve large genomic segments containing additional genes, thus precluding the identification of these Hippo pathway genes as candidate onco-drivers. Indeed, only the deletion of *FAT1* and *WWC1* and amplification of *YAP1* appeared to be highly significant and focal, likely reflecting the specificity and biological impact of their gene copy variations.

Remarkably, both analyses converged in the recurrent and highly significant alterations of *FAT1*, such as somatic mutations and focal gene deletions, in multiple human malignancies (Fig. 1a, b). Some of these frequent genomic alterations in *FAT1* have been recently well documented^12,13^. However, whether FAT1 controls the Hippo pathway is unclear, as its deficiency has been proposed to result in aberrant WNT signaling by increasing the availability of cytosolic β-catenin, similar to classical cadherins^13,14^. In this regard, we found that *FAT1* mutations and gene copy loss occur in many cancers, such as squamous cell carcinomas of the head and neck (29.8%), lung (18.5%), and cervix (9.9%) (Fig. 1b), which are known to retain expression of classical E-cadherin, hence making it improbable that loss of FAT1 will solely contribute to cell–cell contact mediated regulation of β-catenin.

### Coordinated alteration of YAP1 and FAT1 in HNSCC

To begin addressing FAT1 functions in cancer, we focused on HNSCC, the cancer type in which *FAT1* is most frequently mutated (Fig. 1b) and which displays characteristic Hippo signaling dependency, YAP1 overactivity, and frequent *YAP1* gene amplification^9,15^. We posit that these findings may provide an opportunity to investigate the mechanism by which FAT1 regulates YAP in a biologically relevant cancer. The vast majority of *FAT1* mutations in HNSCC are inactivating by virtue of resulting in early termination and likely truncated gene products (Fig. 1c). Moreover, expression of *FAT3* and *FAT4* is limited in most normal oral tissues and HNSCC lesions (Supplementary Fig. 1c). This simplified our analysis by reducing the chance of potential functional compensation between FAT family members, making HNSCC the ideal tumor type to investigate FAT1 function. Remarkably, the use of our recently developed REVEALER^16^ computational approach identified *FAT1* truncating mutations as the top genomic abnormality complementary of *YAP1* gene amplification significantly associated with YAP1 activity (*p* = 0.01), as measured by *CTGF* and *CYR61* overexpression, out of a total of 1589 candidate genomic abnormalities in the large cohort (*n* = 504) of HNSCC (Fig. 1d). This unbiased approach supported the potential role of FAT1 in the regulation of YAP1, and also identified *NOTCH1* mutations and *CYC1* amplification, which achieved nearly statistical significance, as additional candidate YAP1 regulating events in HNSCC.

To begin dissecting the molecular mechanisms controlled by *FAT1* we next studied the gene expression profiles of 264 HNSCC tumors from the TCGA. We stratified tumor cases containing *FAT1* and *FAT2* alterations, including mutations, copy loss alterations or loss of expression (*n* = 188) and compared them to those exhibiting unaltered *FAT1* and *FAT2* (*n* = 76). Analysis of 366 differentially expressed genes between these groups (Supplementary data 1) revealed that the genes upregulated in the *FAT* altered group were enriched for genes containing TEAD2 and TCF4 binding sites in their promoter regions (Fig. 1e and Supplementary data 2), and thus potentially regulated by YAP1 or β-catenin, the latter consistent with prior reports^13,14^. We further confirmed the status of YAP1 protein activation in a panel of 111 primary HNSCC lesions and 5 normal tissues, and observed that the number of cells displaying the active, nuclear localized form of YAP1 positively correlated with disease progression and malignancy. Indeed, in poorly differentiated HNSCC lesions YAP1 activation is generalized (Fig. 1f, g). This trend is similar to that observed in other tumor types such as lung squamous carcinoma, skin melanoma, and cervical cancer (Supplementary Fig. 2), whereas YAP1 activation is absent in most sarcomas and lymphoid tumors, which, coincidentally, display low alteration rates in FAT genes (<5%).

### FAT1 regulates YAP1 in mammals

**Fig. 2:**
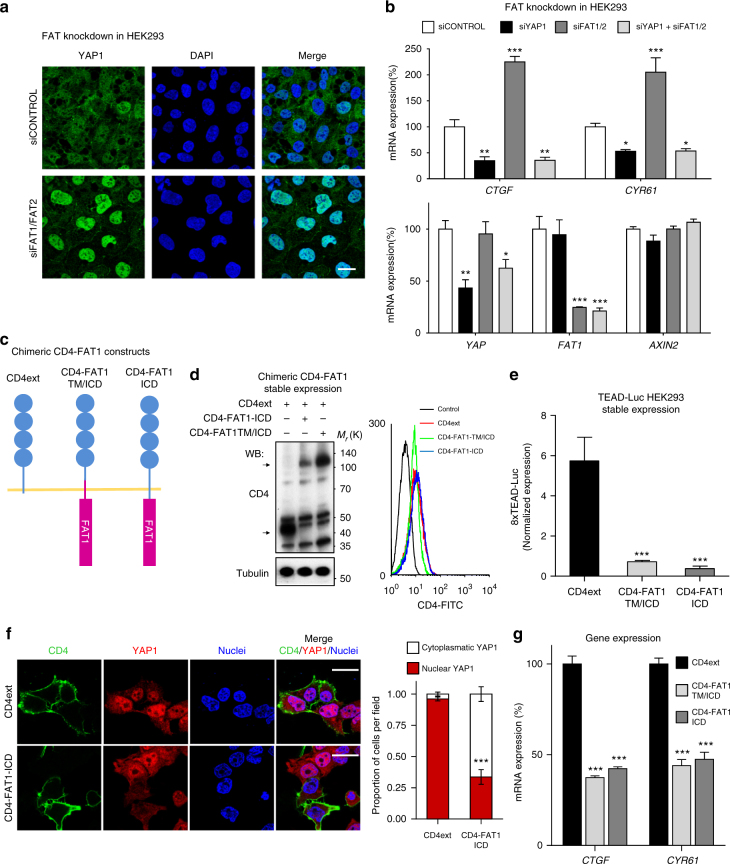
FAT1 regulates YAP1 nuclear localization and activity. **a** siRNA-mediated knockdown of FAT1 and FAT2 in HEK293 cells induces the accumulation of nuclear YAP1 as depicted by immunofluorescence and its associated quantification. In green (Alexa 488), YAP1 staining and in blue, the nuclear counterstain DAPI. A representative experiment is shown. Scale bar, 25 µm. **b** Knockdown of FAT1 and FAT2 induces the upregulation of the YAP1 targets *CTGF* and *CYR61* in HEK293 cells in a YAP1-dependent manner. Gene expression analysis of HEK293 cells transiently transfected with siRNAs against control (scrambled), YAP1, FAT1, and FAT2 as indicated. mRNA levels were evaluated by quantitative PCR (qPCR). Bars represent the GAPDH-normalized mean ± SEM (*N* = 3). **c** Schematic representation of the CD4-FAT1-ICD chimeric constructs. **d** Expression and correct plasma membrane localization of the CD4 chimeras by western blot and FACS. Arrows indicate the specific CD4-chimera bands. Predicted molecular weights CD4ext 46 kDa and both CD4-FAT1 91 kDa**. e** TEAD-Luciferase reporter assay in HEK293 cells stably transfected with the CD4-FAT1 chimeric constructs. Luciferase expression was evaluated in exponentially growing cultures 36 h after transfection. Bars represent mean Renilla-normalized luciferase expression ± SEM (*N* = 4). **f** Transient overexpression of CD4ext and CD4-FAT1-TM/ICD induces YAP1 nuclear exclusion in HEK293 cells 24 h after transfection. A representative immunofluorescence is shown. The nuclear or cytoplasmatic localization of YAP1 was visually evaluated in at least 100 transfected CD4 positive cells from three independent experiments and their quantification is shown in the right panel. Bars represent mean proportion ± SEM of the nuclear and cytoplasmatic localization of YAP1. Scale bar, 20 µm. **g** Quantitative PCR depicting gene expression levels of the YAP1 transcriptional targets *CTFG* and *CYR61* in HEK293 stably expressing CD4-FAT1 ICD constructs. Bars represent the GAPDH-normalized mean ± SEM (*N* = 3). *P < 0.05, **P < 0.01, ***P < 0.001 (One-way ANOVA)

We next explored the potential link between *FAT1* alterations and YAP1 activation using HEK293 cells, a widely used model system to study Hippo signaling and YAP1 function. We began investigating the impact of siRNA-mediated *FAT1* and *FAT2* knockdown, mimicking *FAT1/2* inactivation, and observed a robust YAP1 nuclear translocation (Fig. 2a). This resulted in the *YAP1*-dependent increased expression of the YAP1 target genes *CTGF* and *CYR61*, as it was blunted by YAP1 knockdown (Fig. 2b). In contrast, FAT1/2 knockdown in these cells did not induce the expression of the β-catenin target *AXIN2*^17^. Knockdown of FAT1/2 resulted in Hippo pathway inactivation, as judged by a marked decrease in the levels of phospho-MST1 (pMST1) and phospho-YAP1 (pYAP1) (Supplementary Fig. 3a). We confirmed these findings in human oral keratinocytes (NHOK) isolated from healthy volunteers^18^ (Supplementary Fig. 3b−d). *FAT1/FAT2* knockdown resulted in increased YAP1 nuclear accumulation and increased expression of *CTGF* and to a lesser extent *AXIN2*, supporting a role of FAT1/2 in the regulation of both YAP1 and WNT/β-catenin pathways in these normal cells. FAT1/2 knockdown led to increased growth of these normal epithelial cells, as reflected by their nearly doubled proliferation rate, which was blunted by the concomitant YAP1 knockdown (Supplementary Fig. 3d).

As a complementary approach to knocking down *FAT1/2* expression, we next studied the impact of FAT1 overexpression in HEK293 cells. To overcome the limitation of the sheer length of the *FAT1* gene, as previously noted^12,13^, we used as a molecular tool the expression of the FAT1 intracellular domain (ICD) as this domain has been shown in Drosophila to mediate most of *ft* signaling activity^19^. We engineered chimeric constructs in which the very large extracellular portion of FAT1 was replaced by the extracellular domain of the human CD4 molecule, in which the transmembrane and juxtamembrane regions were provided by either FAT1 (CD4-FAT1-TM/ICD) or CD4 (CD4-FAT1-ICD) (Fig. 2c). These chimeric constructs were correctly expressed and membrane-localized in HEK293 cells, as judged by western blot and FACS analysis (Fig. 2d, and see Fig. 2f). Strikingly, when overexpressed in HEK293 cells, both FAT1 chimeras but not CD4ext control were sufficient to induce the downregulation of a YAP1-regulated TEAD luciferase reporter (Fig. 2e) and nuclear exclusion of YAP1 (Fig. 2f), which is consistently nuclear localized in proliferating, non-confluent HEK293 cells. The inactivation of YAP1 by expression of the CD4-FAT1 chimeric constructs was further confirmed by qPCR analysis of *CTGF* and *CYR61* (Fig. 2g).

### FAT1 forms a molecular complex with Hippo pathway members

**Fig. 3:**
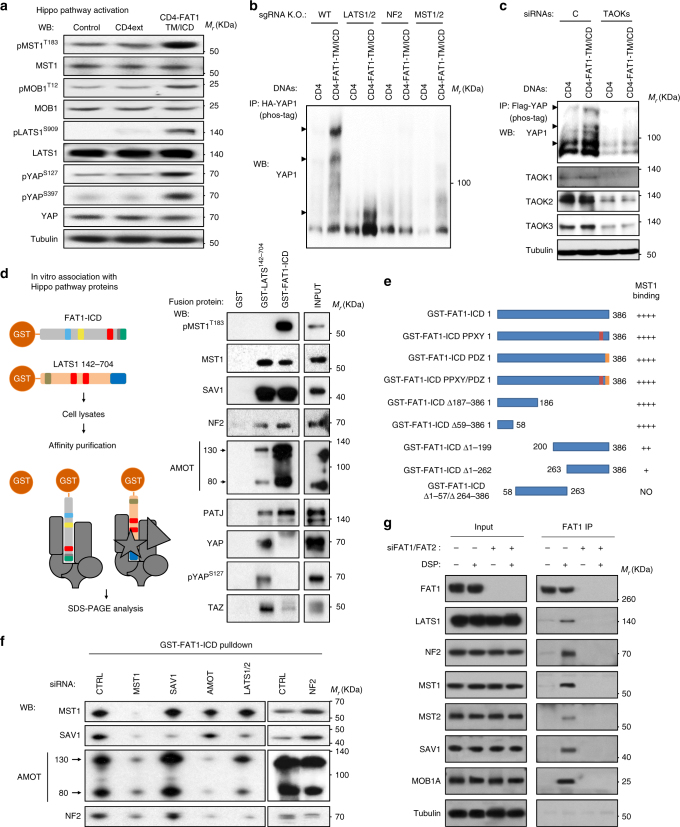
The intracellular domain of FAT1 interacts with and activates the Hippo kinase signalome. **a** Representative western blots against Hippo pathway components in lysates of exponentially growing HEK293 CD4ext and CD4-FAT1-TM/ICD stable cells. Control, parental HEK293 cell line. **b** Analysis of YAP1 phosphorylation after transient transfection with CD4 control or CD4-FAT1-TM/ICD chimera in WT or the corresponding CRISPR/Cas9 sgRNA engineered knockout HEK293 cells as indicated. HA-YAP1 immunoprecipitates were analyzed by phos-tag phosphorylation affinity shift electrophoresis and YAP1 western blotting. Retarded (phosphorylated) YAP1 is indicated by arrowheads. A representative blot is shown. **c** Analysis of YAP1 phosphorylation after transient cotransfection with Flag-YAP1 and CD4 control or CD4-FAT1-TM/ICD chimera in HEK293 cells pretreated with control (C) or TAOK1/2/3 siRNA (TAOKs) as indicated. Flag-YAP1 immunoprecipitates were analyzed by phos-tag electrophoresis and YAP1 western blotting. Retarded (phosphorylated) YAP1 is indicated by arrowheads. A representative blot is shown. **d** On the left, a scheme depicting the GST fusion proteins indicating the approximate location of functional motifs present in FAT1 and LATS1 and the subsequent GST-pulldown assay. On the right, representative western blots of pulldown experiments using GST fusion proteins and HEK293 total cell lysates. **e** Summary of mutant FAT1 ICD constructs (PPXY and PDZ binding site) and the depicted deletions and their ability to bind MST1 as assessed by pulldown assay. **f** siRNA-mediated knockdown on HEK293 of the different components of the Hippo signaling pathway and subsequent GST-FAT1-ICD pulldown on whole cells lysates. **g** Endogenous FAT1 immunoprecipitation by a monoclonal antibody recognizing is extracellular region. Exponentially growing HEK293 were transfected with FAT1 and FAT2 siRNAs for 48 h and then treated for 2 h at 4 °C with DMSO (−) or the reversible crosslinker DSP (+) prior to cell lysis and immunoprecipitation with anti-FAT1. Representative western blots are shown

Because YAP1 nuclear localization is regulated by the activity of the Hippo signaling pathway, we studied the regulation by *FAT1* of the phosphorylation status of the Hippo pathway core components. As shown in Fig. 3a, expression of CD4-FAT1-TM/ICD in HEK293 induced the robust phosphorylation of the kinases MST1 and LATS1, as well as MOB1A and YAP1 without seemingly altering total YAP1 levels, therefore ruling out the degradation of YAP1 as a mechanism by which FAT1 regulates YAP1 activity. To dissect the underlying molecular mechanism, we used a panel of CRISPR/Cas9 engineered HEK293 cells lacking the expression of the critical Hippo pathway components LATS1/2, NF2, and MST1/2^20^. As shown in Fig. 3b, the ability of FAT1 to stimulate YAP1 is strictly dependent on the presence of a completely functional Hippo signaling complex, as removal of any one of its components resulted in the inhibition of pYAP1 accumulation caused by the FAT-1 ICD. While MST1 activation may result from autophosphorylation^21^, recent reports implicating the TAOK1/2/3 kinases in the initiation of the Hippo kinase cascade in Drosophila^22,23^ prompted us to investigate if these kinases play a role in the FAT1-initiated pathway. As show in Fig. 3c, the triple knockdown of TAOK1/2/3 completely impaired YAP1 phosphorylation induced by CD4-FAT1-TM/ICD expression in HEK293 cells, indicating that TAO kinases are strictly required for Hippo pathway activation triggered by FAT1.

We then investigated the ability of FAT1 ICD to physically interact with members of the Hippo pathway by using GST-FAT1-ICD fusion protein purified from bacteria (Supplementary Fig. 4a) to perform pulldown experiments using cellular lysates as indicated in Fig. 3d, using GST-LATS^142–704^ as a positive control. The ICD of FAT1 strongly interacted with MST1, SAV1, NF2, AMOT, and PATJ, which are all key Hippo regulatory components, whereas it did not interact with YAP1, and TAOK-1 (Supplementary Fig. 4b) and had only a very weak interaction with TAZ. The direct interaction of FAT1-ICD with LATS1 was not evaluated in this experiment due to cross-reactivity of the LATS1 antibody with bacterially expressed GST-FAT1-ICD; however, this interaction was demonstrated in cells in vivo (see below). Of interest, FAT1-ICD binds to total and phosphorylated MST1, but GST-LATS^142-704^ failed to interact with pMST1. Deletion mutagenesis analysis of the FAT1-ICD showed that the presence of the first 58 amino acids were sufficient to bind strongly to MST1/2, whereas the last 186 amino acids were able to interact with MST1 independently as well, albeit to a much lesser degree (Fig. 3e). LATS1^142-704^ and FAT1-ICD associated equally well with SAV1, NF2 and PATJ; however in the case of AMOT, it interacted much more strongly with FAT1-ICD. Finally, unlike FAT1-ICD, LATS1^142-704^ interacted strongly with YAP1 and TAZ, and at least a portion of YAP1 was phosphorylated YAP1^S127^. These results indicate that FAT1-ICD can associate with a multimeric complex containing core Hippo signaling components.

To further investigate how the formation of the FAT1-Hippo signaling complex is coordinated, we performed siRNA studies to determine their molecular hierarchy. We successfully knocked down the expression of several key Hippo pathway components (Supplementary Fig. 4c) and studied its impact on complex assembly. As shown in Fig. 3f, MST1 knockdown prevented the efficient interaction of FAT1-ICD with all other members of the complex. On the other hand, LATS1/2 knockdown partially prevented the interaction of FAT1-ICD with SAV1, AMOT and NF2, while AMOT knockdown only affected NF2 binding. Therefore, we concluded that MST1 is an essential component of the complex, likely mediating the direct interaction with FAT1-ICD and promoting the assembly of a multiprotein complex including also LATS, SAV1, NF2, AMOTl, and PATJ (Fig. 3d).

We next sought to confirm the physical interaction of MST1 with FAT1 in vivo. We were unable to consistently detect interaction of CD4-FAT1-TM/ICD with members of the Hippo cascade by standard co-immunoprecipitation and western blotting. We suspected that the failure of FAT1-TM/ICD to co-immunoprecipitate Hippo pathway components was likely due to the labile nature of the FAT-Hippo signaling complexes and their disruption in the presence of the detergent concentrations required for achieving the efficient extraction of CD4-FAT1-TM/ICD from the plasma membrane. Indeed, we readily visualized the interaction of CD4-FAT1-TM/ICD with LATS, NF2, MST1, SAV, and MOB1A upon pretreatment of the cells with a reversible cross linker, dithiobis succinimidyl propionate (DSP), prior to CD4-mediated immunoprecipitation, as shown in Supplementary Fig. 5a. Aligned with our in vitro data, this interaction is dependent on MST1/2, as siRNA-mediated knockdown of MST1/2 readily abolished the formation of immunoprecipitable complexes. Moreover, taking advantage of a monoclonal antibody recognizing FAT1 extracellular region^24^, we observed that endogenous FAT1 associates with the core-Hippo kinase complex, as judged by its co-immunoprecipitation with FAT1 in DSP cross-linked cells, but not after FAT1 knockdown (Fig. 3g). Supporting the role of FAT1 in Hippo complex assembly, endogenous MST1 co-immunoprecipitates with NF2 and LATS/SAV primarily in CD4-FAT1-TM/ICD expressing cells, whereas MST1-MOB1A complex formation does not require FAT1 (Supplementary Fig. 5b). Taken together, these results indicate that the FAT1 ICD interacts with and facilitates the assembly of the core Hippo signaling complex, and that this interaction may induce the activation of Hippo kinases by TAOKs, thereby providing a direct molecular mechanism linking FAT1 to YAP1 phosphorylation and inactivation.

### YAP1 as a molecular therapeutic target in HNSCC

**Fig. 4:**
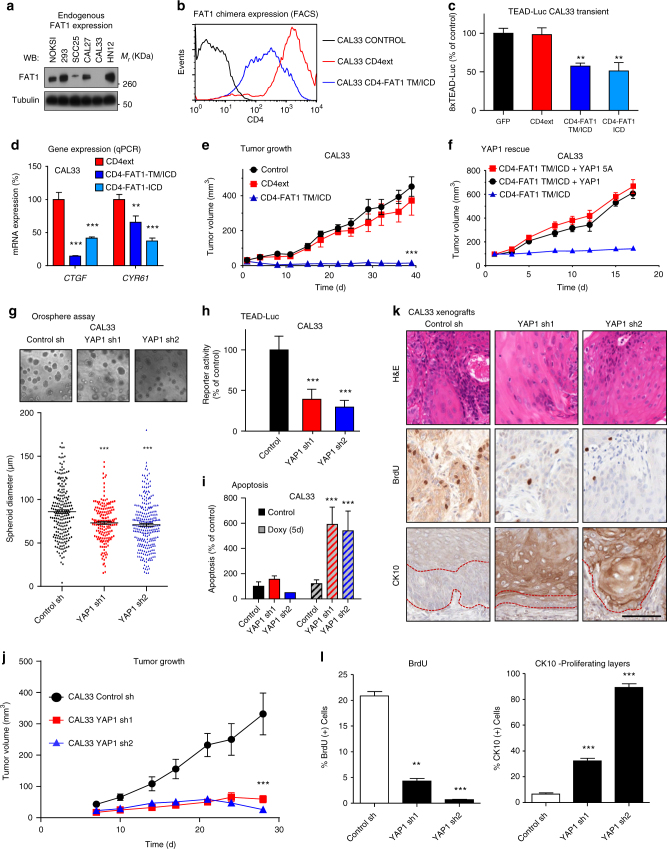
YAP1 is required for HNSCC survival and proliferation in vitro and in vivo. **a** Analysis of FAT1 expression in a panel of epithelial cells, including HNSCC. **b** Expression of CD4ext and CD4-FAT1-TM/ICD chimera in the HNSCC cell line CAL33 by CD4 FACS analysis. **c** TEAD-Luciferase reporter assay in CAL33 cells transiently transfected with the CD4-FAT1 chimeric constructs. Luciferase expression was evaluated in exponentially growing cultures 36 h after transfection. Bars represent mean Renilla-normalized luciferase expression ± SEM (*N* = 4). **d** Quantitative PCR depicting gene expression levels of the YAP1 transcriptional targets *CTFG* and *CYR61* in CAL33 stably expressing the CD4-FAT1 ICD constructs. Bars represent the GAPDH-normalized mean ± SEM (*N* = 3). **e** In vivo xenograft assay. One million CAL33 cells expressing indicated constructs were injected in *nu*/*nu* mice. Data points represent mean volume (*N* = 10 tumors per group) ± SEM. **f** YAP-rescue experiments. In vivo flank xenograft assay as in (**e**) using CAL33 expressing CD4-FAT1-TM/ICD and control or the indicated YAP expression vector. Data points represent mean volume (*N* = 10 tumors per group) ± SEM. **g** Spheroid formation assay of stable CAL33 shRNA control and *YAP1* shRNA cell lines. Representative pictures are shown on top and diameter quantifications (>200 colonies per group) are shown below. Black lines represent mean ± SEM. **h** CAL33 stably expressing control and *YAP1* shRNAs were stimulated with doxycycline for five days (1 µg/ml) and then transfected with a 8xTEAD-luciferase reporter. Renilla-normalized reporter activity is expressed as % of control. Bars represent mean ± SEM (*N* = 4). **i** Apoptosis assay by propidium iodide staining of CAL33 cell lines expressing control or *YAP1* shRNA after 5d of Doxycycline stimulation. Bars represent mean ± SEM (*N* = 4). **j** In vivo xenograft assay. One million cells were injected in *nu/nu* mice. Animals were fed Doxycycline food (6 g/Kg) *ad libitum* 24 h h after tumor cell injection for the duration of the experiment. Data points represent mean volume per group (*N* = 10 tumors) ± SEM. **k** Representative immunohistochemical stainings of CAL33 tumors from panel (**j**). In the Cytokeratin 10 (CK10) panels the dotted red line delimits the proliferating front of the tumor. Scale bar, 100 µm. **l** Automated histological quantification of stainings in (**k**), bars represent mean ± SEM (*N* = 3). **P < 0.01, ***P < 0.001 (One-way ANOVA)

To explore whether FAT1 alterations and its associated downstream molecular signaling play a role in tumorigenesis we used representative HNSCC-derived cells displaying prototypical FAT1 alterations as evidenced by exome sequencing of a large cell panel^25^, and identified SCC25 (FAT1 copy loss), CAL27 (heterozygous FAT1 copy loss), and CAL33 (hemizygous FAT1 K3504X mutation and loss of the remaining allele) as displaying abnormal levels of FAT1 protein compared to NOKs exhibiting wild-type FAT1 (Fig. 4a). We sought to restore FAT1 function in tumorigenic HNSCC cells (CAL33 and CAL27) by generating stable cell lines expressing the CD4-FAT1-TM/ICD chimeric construct or its control (CD4ext). The appropriate expression and localization of these constructs was confirmed by FACS (Fig. 4b and Supplementary Fig. 6b) and resulted in decreased YAP1 activity as depicted by reporter assay (Fig. 4c) and qPCR of the *CTGF* and *CYR61* transcriptional targets (Fig. 4d). Remarkably, expression of CD4-FAT1 ICD, but not its controls, displayed reduced cell proliferation (Supplementary Fig. 6a) in vitro and abolished tumorigenesis in vivo (Fig. 4e and Supplementary Fig. 6c). These transcriptional and biological effects of CD4-FAT1-TM/ICD were both rescued by overexpression of YAP1 or its mutant that cannot be phosphorylated by LATS (Fig. 4f and Supplementary Fig. 6d, e), supporting that YAP1 function is tightly regulated by FAT1.

As FAT1 mutations may ultimately render cancer cells dependent on YAP1 function, we next knocked down YAP1 expression using tetracycline-inducible YAP1 shRNA lentiviruses in CAL27 and CAL33 cells, and in tumorigenic HN12 that display normal FAT1 levels (Fig. 4a) but harbor YAP1 gene copy gain^25^. Successful knockdown of YAP1 was confirmed by western blot (Supplementary Fig. 6f and 8a). Knockdown of YAP1 resulted in significantly smaller spheroid growth in these HNSCC cells (Fig. 4g, and Supplementary Fig. 8b), reduced GAL4-TEAD reporter expression (Fig. 4h and Supplementary Fig. 6g and 7c) and 3–5-fold increase in basal apoptosis (Fig. 4i and Supplementary 6h). We evaluated the impact of YAP1 downregulation in vivo in a tumor xenograft model. As shown in Fig. 4j and Supplementary Fig. 6i and 7d, two independent YAP1 shRNAs completely abrogated tumor growth in CAL33 and CAL27 cell lines, and strongly inhibited tumor growth of HN12 HNSCC cells, which correlated with decreased proliferation as depicted by BrdU incorporation (Fig. 4k, l, Supplementary Fig. 6j), and in some cells (CAL33), increased expression of the epithelial differentiation marker Cytokeratin 10 (CK10) (Fig. 4k, l).

**Fig. 5:**
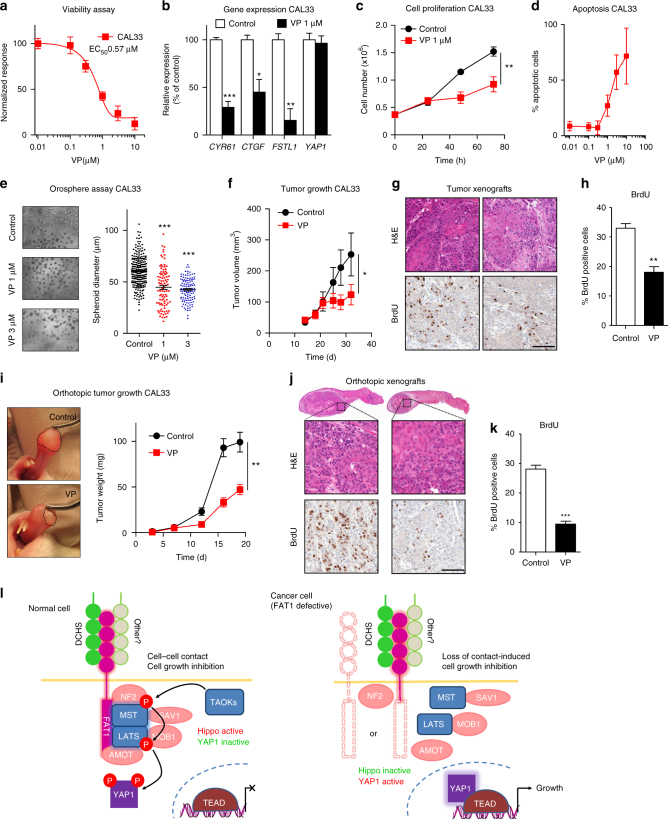
The YAP1 inhibitor Verteporfin (VP) impacts cell survival, proliferation, and tumor growth. **a** Dose-response experiment for cell viability as determined by the AlamarBlue assay in CAL33 cell subjected to 48 h treatments with VP. Data points represent mean ± SEM (*N* = 8). **b** Gene expression determination by quantitative PCR of YAP1 target genes *CYR61*, *CTGF*, and *FSTL1* after 18 h treatment with 1 µM VP. Bars represent mean ± SEM (*N* = 4). **c** Proliferation assay by cell counting of CAL33 cells exposed to vehicle (Control) or 1 µM VP for the times indicated. Data points represent mean ± SEM (*N* = 4). **d** Apoptosis assay by propidium iodide staining. Dose-dependent VP-induced apoptosis at 48 h in CAL33 HNSCC cell. Data points represent mean ± SEM (*N* = 3). **e** Spheroid formation assay of CAL33 cells treated with VP as indicated for 10 d. Representative pictures are shown on top, and diameter quantifications (>95 colonies per group) are shown below. Black lines represent mean ± SEM. **f** In vivo tumorigenesis assay in *nu*/*nu* mice. One million CAL33 cells were injected s.c. and tumors were allowed to grow until ~100 mm^3^. Before treatment the animals were randomized so that the mean tumor size per group was as equal as possible between groups at the initiation of treatment. Then VP was injected daily i.p. (50 mg/Kg). Data points represent mean tumor volume ± SEM (*N* = 10). **g** Representative immunohistochemical stainings of CAL33 tumors from panel (**f**). Scale bar, 100 µm. **h** Automated histological quantification of BrdU staining in (**g**). Bars represent mean ± SEM (*N* = 5). **i** In vivo tumorigenesis assay by oral orthotopic injection in SCID/NOD mice. Fifty thousand CAL33 cells were injected in the tongue, while VP was injected daily i.p. (50 mg/Kg) starting 24 h after implantation for the duration of the experiment. Right panel, representative gross appearance of the tumor lesions at collection time. Left panel, data points represent mean tumor weight ± SEM (*N* = 10). **j** Representative immunohistochemical stainings of CAL33 tumors from panel i. Scale bar, 100 µm. **k** Automated histological quantification of BrdU staining in (**j**). Bars represent mean ± SEM (*N* = 5). *P < 0.05, **P < 0.01, ***P < 0.001 (One-way ANOVA). **l** Scheme depicting the proposed molecular mechanism model, see text for details

The dramatic impact of YAP1 knockdown in HNSCC tumorigenesis prompted us to evaluate the possibility of the pharmacological intervention on YAP1 as a therapeutic venue in HNSCC. Although there are not yet specific YAP1 inhibitors, the U.S. Food and Drug Administration (FDA) approved drug Verteporfin (VP) was identified as a disruptor of the interaction between YAP1 and the TEAD2 transcription factor in a small molecule inhibitor screen, thereby inhibiting YAP1 signaling output and reverting the impact of *YAP1* overexpression or *NF2* inactivation in vivo^26^. First, we evaluated the effect of VP on cell viability in vitro and determined that the IC_50_ for CAL27, CAL33, and HN12 is in the low µM range (Fig. 5a and Supplementary Fig. 8a and 8d) aligned with its previously reported activity^26^. This correlated with a reduction in the mRNA levels of the YAP1 transcriptional targets *CYR61*, *CTGF*, and *FSTL*, while *YAP1* mRNA levels remained unaffected and hence served as a control (Fig. 5b and Supplementary Fig. 8e), supporting the idea that VP causes the inhibition of YAP1 transcriptional activity. Moreover, VP reduced the proliferation of HNSCC and tumor spheroid formation in vitro (Fig. 5c, e, and Supplementary Fig. 8b and 8f) and caused apoptosis in HNSCC cells in vitro (Fig. 5d and Supplementary Fig. 8c). We then evaluated the impact of VP in vivo in HNSCC cells in flank and oral orthotopic xenograft models. In both cases, VP reduced tumor growth and cancer cell proliferation when administered daily (Fig. 5f–k), suggesting that YAP1 is an attractive therapeutic target in human HNSCC and likely other human malignancies.

## Discussion

The recent advance on massively parallel sequencing techniques has afforded an unprecedented wealth of information about the genetic makeup of human cancer. The emerging information confirmed the preponderant roles of many classical tumor suppressors and oncogenes while some other less studied molecules have been brought under the spotlight. Using HNSCC as an example, this cancer type displays widespread genetic alterations that include inactivating mutations of the tumor suppressor *TP53* (70% of the cases) and mutations, homozygous deletions and promoter methylation of *CDKN2A* (49%), which are believed to be prerequisites for tumor formation^27^. This requirement of tumor suppressor inactivation can be bypassed by HPV infection^27,28^. Recurrent mutation and amplification of *PIK3CA* (35%) and to a lesser extent *HRAS* (4%) mutations have emerged as the most frequent oncogenic drivers in both HPV- and HPV + HNSCC, with other potential oncogenic events including *EGFR*, *CCND1* overexpression, or *PTEN* inactivation^27,28^. Here, we provide evidence that inactivation mutations and genomic alterations in *FAT1* results in YAP1 activation via inactivation of the Hippo signaling pathway, thereby contributing to the progression of HNSCC and likely multiple other human malignancies displaying unrestrained YAP1 function.

Our analysis identified genomic alterations in members of the canonical Hippo pathway, including *LATS1* and *NF2* in some cancers, whose potential role in YAP activation warrants further investigation in each individual cancer type. Remarkably, *FAT1* mutations and focal gene deletions resulting in the loss of its protein product appear to be widespread. However, whether human FAT1 regulates the Hippo pathway has been much less clear, and if so its underlying mechanism was not known. Most prior studies on YAP1 regulation in mammals have focused on the role of *FAT4* due to its genetic relatedness to drosophila *ft*^19^, but the role of FAT1 and other FAT members in mammals during development is less dramatic than ft in drosophila. In this regard, it is now evident that the regulation of the Hippo pathway has diverged from arthropods to vertebrates, likely to accommodate the unique developmental, physical and physiological features of both phyla^5^. FAT1 regulates the development and function of certain organs such as the kidney, while knockdown experiments in mice and rat suggest that FAT1 has an important role in the development of the central nervous system (CNS)^29^, and acts upstream of TAZ in neural differentiation^30^. Because alterations in *FAT1* KO mice occur with a variable penetrance, it is possible that other FAT members may compensate for *FAT1* loss, particularly in tissues such as the CNS during development, in which all FAT family members are consistently expressed^31^, likely precluding the display of stronger phenotypes. Indeed, our analysis identified MST/Hippo binding sites in FAT1 ICD in areas that are conserved in other FAT members, thus providing a potential structural basis for their redundancy. As FAT expression levels drop in adult tissues^32^, it is possible to hypothesize that this mechanistic compensatory effect might be reduced in cancers arising during adulthood. For example, disruption of FAT1 function could be sufficient to dysregulate Hippo signaling in the squamous epithelium, as this tissue expresses FAT3 and FAT4 poorly and FAT2 might not be sufficient to compensate FAT1 deficiency fully.

Overall, our oncogenomic and functional analyses support the role of FAT1 in cancer as a tumor suppressor acting upstream of YAP1. Of interest, this raises the possibility that FAT1 may control β-catenin, as previously reported^13^, and YAP1 independently. Alternatively, FAT1 deficiency may promote β-catenin activation indirectly through a recently described mechanism downstream from YAP1^33^. Further studies re-expressing full length FAT1 and its deletion mutants in FAT1 defective cells may facilitate addressing these exciting possibilities, as well as extend our analysis using FAT1 ICD chimeras. Mechanistically, we show that chimeric and endogenous FAT1 signals to YAP1 by its direct interaction with MST1 and the consequent association with a multimeric signaling complex including the core Hippo pathway kinases and their regulatory and scaffolding molecules, such as AMOT and NF2. This, in turn, may result in the activation of MST1 by TAOKs and the subsequent phosphorylation of LATS and YAP1 (Fig. 5l). How precisely FAT1 association to MST1 may lead to its phosphorylation by TAOKs is not fully understood. We can speculate that the physical interaction between MST1 and FAT1 and the assembly of the Hippo kinase complex at the plasma membrane may relieve MST1 from an auto inhibitory mechanism^21^, thereby enabling its recognition and phosphorylation by TAOKs, ultimately leading to the activation of the Hippo kinase cascade (Fig. 5l). This and other possibilities by which FAT1 may regulate YAP, including the recruitment of TAOKs to the proximity of MST1 by additional yet to be identified Hippo signaling complexes, or FAT1 and TAOK acting on other Hippo pathway components warrant further investigation.

The high frequency of alterations in the *FAT* gene family can now provide a widespread mechanism contributing to the recurrent activation of YAP1/TAZ in human malignancies, in addition to other more restricted processes in other tumor types^34–36^ (Fig. 5l). In turn, targeting YAP1/TAZ function or their relevant downstream targets poses a very attractive therapeutic venue that also justifies further investigation. In this regard, while VP cannot be considered a specific YAP1 inhibitor and is likely to impact several other molecular targets in addition to blocking TEAD-YAP1 interactions^37,38^, this FDA-approved agent decreased the expression of YAP1-regulated genes and its administration resulted in increased apoptosis, and decreased proliferation and HNSCC tumorigenesis in vivo, mimicking YAP1 knockdown in these cells. These observations provide further support of the potential benefits of the use of pharmacological inhibitors of YAP1 in cancer. Specifically for HNSCC, our results indicate that the frequent alteration of *FAT1* might result in defective Hippo signaling and unrestrained YAP1 activity, thus raising the possibility that molecular therapies targeting YAP1 or its downstream targets may represent an attractive precision medicine therapeutic option for the treatment of this and other malignancies harboring genomic alteration in the *FAT* family of tumor suppressor genes.

## Methods

### Antibodies and reagents

Antibodies against YAP1, pMST1^T183^, MST1, pMOB1^T12^, MOB1, pLATS1^S909^, LATS1, LATS2, AMOT, NF2, SAV1, PATJ, pYAP^S127^, pYAP^S397^ (1:2000), and Tubulin-HRP (1:10000) were purchased from Cell Signaling Technology (Beverly, MA). A monoclonal antibody against FAT1 (NTD14, 1:1000) against the *nz*-terminal region has been recently described^24^. Cytokeratin 10 rabbit polyclonal antibody was purchased from Covance (Princeton, NJ) (1:1000), anti-BrdU (Bromodeoxyuridine) Rat monoclonal antibody was from Accurate (Westbury, NY). Anti-TAZ antibody was purchased from Santa Cruz (Santa Cruz, CA)(1:2000). Antibodies anti-CD4 (OKT4) was from eBioscience (San Diego, CA)(1:2000). Verteporfin (VP) was purchased from USP (Rockville, MD).

### Cell lines, culture conditions and transfections

CAL27, CAL33, and HN12 cell lines were obtained from the NIDCR Oral and Pharyngeal Cancer Branch cell collection^25^. Their identity was confirmed by STR profiling and were tested free of mycoplasma infection. HEK293 were purchased from ATCC (Manassas, VA). CAL27, CAL33, HN12, and HEK293 cell lines were cultured on DMEM (D-6429, Sigma-Aldrich, St. Louis, MO), 10% fetal bovine serum, 5% CO2, at 37 °C. Normal Human Oral Keratinocytes (HNOK) lines were prepared as described before^39^ and grown on Defined Keratinocyte-SFM with supplements and antibiotics (Life Technologies, Carslbad, CA). LATS1/2-, MST1/2-, or NF2-deficient HEK293A cells were created through the CRISPR/Cas9 system as described before^20^. SiRNAs SMARTpool ON-TARGETplus for FAT1, FAT2, SAV1, NF2, non-targeting control, and YAP1-inducible lentiviral shRNAs, clones V2THS_65509 and V2THS_247011 were from GE Healthcare (Lafayette, CO). siRNAs for MST1 were purchased from IDT (DsiRNA Duplexes human MST1 5′-AGUUGUCGCAAUUAAACA 5′-AGGUACUUGUUUAAUUGC, IDT, Coralville, IA). SiRNAs for LATS1 and LATS2 were from Sigma (MISSION siRNA human LATS1 SASI_Hs01_00046128, siRNA human LATS2 SASI_Hs01_00158803)^40^.

### Immunohistochemistry

H&E stained paraffin sections were used for histopathological evaluation. For immunohistochemistry, 5-μm unstained paraffin sections were deparaffinized in three changes of SafeClear II (ThermoFisher Scientific, Waltham, MA), 5 min each, and the hydrated with graded alcohols (100°, 95°, 70°), two changes each, 5 min each. The endogenous peroxidase was blocked by incubating for 30 min in 3% H2O2 in 70° ethanol. Antigens were retrieved with 10 mM citric acid (2.1 g/L) in a microwave oven, 2 min at 100% power, followed by 18 min at 20%. The slides were allowed to cool for 15 min and washed extensively with distilled water, followed by three changes of PBS, 5 min each. After blocking with 2.5% BSA in PBS at room temperature, for 30 min, the slides were incubated overnight at 4 °C with the appropriate primary antibodies diluted in 2.5% BSA in PBS. The slides were then washed with PBS, 3 × for 5 min, and successively incubated biotinylated anti-rabbit/rat immunoglobulins, 1:400 in blocking buffer at room temperature, for minutes, washed with PBS 3 × for 5 min each, and incubated with ABC complex (Vector Labs, Burlingame, CA), 30 min at room temperature. The slides were extensively washed with PBS; the reaction was developed with 3,3′-Diaminobenzidine under microscopic control and stopped with distilled water. The slides were the counterstained with Hematoxylin and washed 15 min in running tap water to bluish, dehydrated in graded alcohols (70°, 95°, 100°), cleared in SafeClear II and mounted in Permount mounting media (ThermoFisher Scientific). The histological slides were processed and developed at the same time to minimize inter-assay variability. All stained slides were scanned at ×40 using an Aperio CS Scanscope (Aperio, Vista, CA) and quantified using the available Aperio algorithms.

### Immunofluorescence and image quantification

NOK were seeded on the coverslips coated with collagen, 293 cells were seeded on coverslips coated with poly-D lysine (Sigma-Aldrich, St. Louis, MO). For immunofluorescence, the cells were washed with ice-cold PBS and fixed with 3.2% paraformaldehyde in PBS. Then the cells were washed three times with PBS and permeabilized with Triton X-100 0.1% in glycine 200 mM in PBS. Nonspecific binding was blocked with 3% of bovine serum albumin (BSA) in PBS for 1 h. Fixed cells were incubated with the primary antibody overnight at 4 °C, followed by 1.5 h incubation with the secondary antibody. Nuclei were stained with Hoechst 33342 (Life Technologies). Images were taken using Zeiss Axio Imager Z1 microscope equipped with an Apotome device (Carl Zeiss, Peabody, MA) and a motorized stage. Final images were bright contrast adjusted with Zen 2012 (Carl Zeiss) or PowerPoint. Image quantifications were performed with ImageJ with the MBF ImageJ bundle (http://www.macbiophotonics.ca/imagej/installing_imagej.htm). For nuclear YAP the intensity per nucleus was quantified by drawing a region of interest around nuclei and calculating the average gray value per nucleus.

### Quantitative PCR

RNA was extracted from exponentially growing cultures by the TriZol method following manufacturer’s recommendations (Life Technologies). Samples were excluded in cases were mRNA quality after processing was poor (RIN < 7). One microgram total RNA was converted to cDNA using the Suprescript III kit (Invitrogen). One microgram cDNA was used as input for the PCR reactions. GAPDH was used for normalization.

Primers: FAT2 (Hs.PT.58.39666826), AXIN2 (Hs.PT.58.39305692). PrimeTime qPCR assays were purchased from Integrated DNA Technologies (Coralville, IA). A list of the primer sequences used for qPCR is present in the Supplementary data 3.

### Western blotting

Exponentially growing cells were washed in cold PBS, lysed on ice in RIPA buffer (0.5% NaDOC, 0.1% SDS, 25 mM Hepes pH 7.5, 100 mM NaCl, 1.5 mM MgCl2, 0.2 mM EDTA, 1% Triton X-100, 20 mM β-glycerophosphate, 0.5 mM DTT, 1 mM phenylmethylsulfonyl fluoride [PMSF], 1 × Complete Mini Protease Inhibitor Cocktail (Roche, Indianapolis, IN)), and cell extracts collected, sonicated, and centrifuged to remove the cellular debris. Supernatants containing the solubilized proteins were quantified using the detergent compatible DC protein estimation kit (Bio-Rad, Hercules, CA); equal amounts by mass were separated by SDS-PAGE, and transferred to PVDF membranes (Millipore Corporation, Billerica MA). Equivalent loading was confirmed with Ponceau-S staining. For immunodetection, membranes were blocked for 1 h at room temperature in 5% non-fat dry milk in T-TBS buffer (50 mM Tris/HCl, pH 7.5, 0.15 M NaCl, 0.1% [v/v] Tween-20), followed by 2 h incubation with the appropriate antibodies, in 1% BSA-T-TBS buffer. Detection was conducted by incubating the membranes with horseradish peroxidase–conjugated goat anti-rabbit IgG secondary antibody (Southern Biotech, Birmingham, AL, USA) at a dilution of 1:50,000 in 5% milk-T-TBS buffer, at room temperature for 1 h, and visualized with Immobilon Western Chemiluminescent HRP Substrate (Millipore). Uncropped versions of the western blots in the main figures are shown in the Supplementary Fig. 9−11.

### Cytometry

For apoptosis determination, the cells were trypsinized, washed once with PBS, and fixed in 50% ethanol for 30 min at 4 °C. After fixation, the cells were centrifuged, resuspended in a 1.12% sodium citrate, and 10 µg/mL RNase A in PBS and incubated at 37 °C for 30 min. Propidium Iodide (PI) was added to a final concentration of 50 µg/mL 5 min before the analysis. Samples were analyzed in a FACScalibur cytometer (Becton Dickinson, Franklin Lakes, NJ). For anti-CD4 staining, the cells were collected by incubation on PBS/EDTA/FBS (2 mM EDTA and 0.005% FBS in PBS) until detached. The cells were washed once in PBS/BSA (3% BSA in PBS), resuspended in blocking buffer (3% mouse serum in PBS) and incubated for 20 min on ice. FITC-conjugated anti Human CD4 antibody (BD Biosciences, San Jose, CA) was added (1:100) and incubated for 30 min on ice. The cells were washed once with PBS/BSA and immediately analyzed in and FACScalibur cytometer.

### Reporter assays

HEK293 cells were transfected in 24-well plates with 1 µg the CD4 chimeric expression vectors in combination with 100 ng of a 8 × TEAD Firefly luciferase reporter (Addgene ID 34615, Addgene, Cambridge, MA), 100 ng of a Pol III-driven Renilla Reniformis Luciferase for normalization (pRL U6) and 100 ng of pCEFL mCherry, an EF-1α driven mCherry expression vector to monitor transfection efficiency. Luciferase activity was measured 24 h after transfection in a Biotek Synergy Neo luminometer (Winooski, VT).

### GST Pulldowns

GST-LATS1-142-704 was obtained by PCR amplification from the vector p2xFlag CMV2 LATS1 (a gift from Marius Sudol) and cloned into pGEX4T3 with adapters for *Bam*HI and *Not*I. GST-FAT1-ICD was generated by subcloning FAT1-ICD from pCEFL CD4-FAT1-ICD into the *Bam*HI and *Not*I sites of pGEX 4T3. Both GST fusion proteins, and GST as a control, were expressed in BL21 *E.coli* transfected with each pGEX4T3 construct and coupled to GST-Sheparose beads. To incubate the GST beads with mammalian cell extracts, HEK293 cells were cultured in 15 cm plates and, upon reaching confluency, they were quickly washed twice in ice-cold PBS and then lysed in pulldown buffer (1 mL of 50 mM HEPES (pH 7.5), 150 mM NaCl, 10% glycerol, 0.3% (w/v) CHAPS, 1.5 mM MgCl_2_, 1 mM EGTA, 100 mM NaF, 500 μM sodium orthovanadate, 10 μg/mL aprotinin, and 10 μg/mL leupeptin and 1 mM PMSF). The pulldown was performed by adding 20 µL of beads (or 10 µg of protein) to 1 mL of cell lysate and incubated at 4 °C for 2 h with their corresponding GST beads (Amersham, Piscataway, NJ). Beads were then washed three times with pulldown buffer, resuspended in Laemmli sample buffer and resolved by SDS-PAGE.

### Immunoprecipitation, kinase assay, and crosslinking

Exponentially growing cultures in 150 mm plates were lysed in CHAPS buffer (1 mL of 50 mM HEPES (pH 7.5), 150 mM NaCl, 10% glycerol, 6 mM CHAPS, 1.5 mM MgCl_2_, 1 mM EGTA, 100 mM NaF, 500 μM sodium orthovanadate, 10 μg/mL aprotinin, and 10 μg/mL leupeptin and 1 mM PMSF). A volume of 15 µL of anti-CD4 antibody (OKT4 clone, eBiosciences) and 20 µL of Gammabind G Sepharose beads (GE Healthcare) were premixed in 500 µL CHAPS buffer and then combined with the lysate and incubated on a rocker for 2 h at 4 °C. Beads were washed four times with CHAPS buffer, once in wash buffer (40 mM HEPES, 200 mM NaCl), and once in kinase assay buffer (30 mM HEPES, 50 mM potassium acetate, 5 mM MgCl_2_). The immunoprecipitate was subjected to a kinase assay for 30 min at 30 °C in the presence of 200 µM ATP and 100 ng of GST-MOB1A beads expressed and purified in *E. coli* BL21 cells. Kinase reactions were stopped by resuspension in Laemmli sample buffer and resolved by SDS-PAGE. Crosslinking experiments were performed as described elsewhere^41^, differing in the immunoprecipitation step that was performed as above.

### Spheroid formation

Spheroid (orosphere) assays were performed in 24-well plates. Briefly, the wells were coated with 250 µL, 1.5%, agarose in DMEM. Ten thousand cells were resuspended in 0.5 mL warm DMEM, 10% FBS, and 0.1% agarose containing the appropriate treatment and layered on top. Agarose was allowed to solidify at room temperature for 15 min before adding an additional 0.5 mL of DMEM, 10% FBS containing the same treatment as above, and returning the cells to the incubator. The cells were allowed to grow for two weeks, replacing the liquid media every three days before spheroid formation was analyzed. For quantification, the wells were photographed using an automatic whole well scan mode in a Zeiss Axiovert 200 M microscope. Colony counting and diameter measurements were performed manually using the Zeiss Axiovision 4.8 software.

### Animal work

All animal studies were carried out according to NIH-approved protocols, in compliance with the Guide for the Care and Use of Laboratory Animals (http://www.iacuc.org/) and approved by the University of California San Diego (UCSD) Institutional Animal Care and Use Committee (IACUC). All handling, transplantation, and infection procedures were conducted in a laminar-flow biosafety hood. Female athymic (nu/nu) nude mice (Harlan Sprague-Dawley, Frederick, MD), 5–6 weeks of age and weighing 18–20 g, were used in the study, and housed in appropriate sterile filter-capped cages and fed and watered ad libitum. No randomization was used prior to experimental group assignment except when indicated. Briefly, exponentially growing HNSCC cultures were harvested, washed, resuspended in DMEM, and 1 × 10^6^ viable cells were transplanted s.c. into both flanks of the athymic mice. For orthotopic injections, SCID/NOD 5–6 weeks of age female mice were used. Briefly, 1 × 10^5^ resuspended in 50 µl of DMEM were used and injected in the tongue from the ventral aspect of the tongue following a posterior to anterior trajectory of the needle, so that the cells accumulate toward the tip of the tongue. The investigators were not blinded to allocation of samples during experiments and outcome assessment. For tumor growth analysis, tumor weight was determined, whereby tumor volume ((LW2/2); were L and W represent the length and the width of the tumor) was converted to weight (mg) assuming unit density. No statistical method was used to estimate animal sample size. The animals were monitored twice weekly for tumor development;^42^. Results of animal experiments were expressed as mean ± SEM, and unpaired Student’s *t*-test was used to determine the difference between experimental and control groups for each of the transplanted cell lines. *p* < 0.05 was considered to be statistically significant.

### Proliferation and viability

Population doubling was calculated as described elsewhere^43^. For viability assays, cells in either 96 well plates were treated as indicated and supplemented with 1/10 of the culture volume of AlamarBlue (Invitrogen, Carlsbad, CA) reagent for the last 6 h of the treatment. Absorbances were recorded at 570 nm in a Biotek Synergy Neo microplate reader^44^.

### Genomic data analysis

Gene mutation and copy number variation analyses were performed using publicly available data generated by The Cancer Gene Atlas (TCGA) consortium^9^, accessed through the UCSD Cancer Browser (genome-cancer.ucsc.edu), the Memorial Sloan Kettering Cancer Center cBIO portal (www.cbioportal.org), Broad’s Institute Firehose GDAC (gdac.broadinstitute.org/), GISTIC (www.broadinstitute.org/tcga), and MutSigCV^10^ (www.tumorportal.org). The copy number data for Hippo pathway genes in the TCGA pancancer was obtained from the TCGA Copy Number Portal at the Broad Institute (http://portals.broadinstitute.org/tcga/home). The mutational significance of Hippo pathway genes in the TCGA pancancer dataset was obtained from the pancancer MUTSIG results in the supplementary data 3 of Lawrence et al. 2013. The individual TCGA cohort MUTSIG significance was obtained from FireBrowse release 20160128 (firebrowse.org). Gene expression analysis was performed with the Partek Genomics Suite version 6.6 (Partek, St. Louis, MO). Gene enrichment analysis was performed using the Enrichr software^45^. Custom python code used during the TCGA analysis is available upon request.

### REVEALER analysis

The REVEALER analysis finds mutually exclusive genomic variants that correlate with a “functional” phenotype such as the activation of YAP1^16^. We used mutations and copy number alterations from the level 3 TCGA HNSCC data from the Firehose Portal (https://gdac.broadinstitute.org/). The REVEALER analysis included pre-filtered variants from the mutation and copy number HNSCC TCGA datasets (a total of 1589 candidate genomic abnormalities). The functional target for REVEALER was the mean normalized expression of CTGF and CYR61 from the TCGA HNSCC RNASeq dataset. As the input “seed” to REVEALER we used the amplification status of YAP1 (YAP1_AMP).

### Statistical analysis

No statistical method was used to predetermine sample size. The investigators were not blinded to allocation of samples during experiments and outcome assessment. All analyses were performed in triplicate or greater and the means obtained were used for ANOVA or independent, two-tailed, unpaired *t*-tests. Statistical analyses, variation estimation, and validation of test assumptions were carried out using the Prism 6 statistical analysis program (GraphPad). Asterisks denote statistical significance (non-significant or N.S., *p* > 0.05; *p < 0.05; **p < 0.01; and ***p < 0.001). All data are reported as mean ± standard error of the mean (S.E.M).

### Data availability

All data supporting the findings in this study are available from the corresponding author upon reasonable request.

## Electronic supplementary material

Supplementary Information

Peer Review File

Description of Additional Supplementary Files

Supplementary Data 1

Supplementary Data 2

Supplementary Data 3

Supplementary Data 4
