# Supplementary material for: Assembly and activation of the Hippo signalome by FAT1 tumor suppressor
Source: Nat Commun. 2018 Jul 9;9:2372. doi: 10.1038/s41467-018-04590-1 (PMC6037762; doi:10.1038/s41467-018-04590-1)

Supplementary Figure 1

a **Hippo signaling alterations in cancer**

Gene	Pan-Cancer *MUTSIG Significant*	CNV Type	*GISTIC q-value*
AMOT	NO	–	NS
AMOTL1	NO	–	NS
AMOTL2	NO	–	NS
DCHS1	NO	D	1.68E-28
DCHS2	NO	D	7.00E-15
FAT1	YES	D	2.65E-117
FAT2	NO	–	NS
FAT3	NO	–	NS
FAT4	NO	D	0.23
FRMD6	NO	–	NS
LATS1	NO	D	9.69E-28
LATS2	NO	D	2.12E-38
NF2	NO	–	NS
SAV1	NO	–	NS
STK3	NO	A	2.04E-09
STK4	NO	–	NS
TEAD1	NO	D	6.94E-07
TEAD2	NO	D	2.90E-15
TEAD3	NO	–	NS
TEAD4	NO	A	1.71E-15
WWC1	NO	D	8.33E-03
WWTR1	NO	A	4.70E-16
YAP1	NO	A	6.66E-03

b **Cancer-specific mutations in Hippo signaling genes**

Gene	Cancer type (*MUTSIG Significance)*	
AMOT	COAD (4.97e-03)	COADREAD (4.06e-04)
FAT1	BLCA (1.16e-04)	HNSC (1.44e-11)
	KIPAN (7.10e-02)	UCEC (6.62e-03)
FAT2	CESC (5.21e-02)	
FRMD6	COAD (6.26e-02)	
LATS1	COAD (1.68e-03)	COADREAD (3.15e-03)
	LIHC (1.36e-01)	PAAD (1.41e-01)
NF2	COAD (3.85e-06)	COADREAD (1.93e-06)
	KIPAN (1.55e-09)	KIRC (9.51e-03)
	KIRP (8.99e-06)	PAAD (3.46e-06)
SAV1	KIRP (1.33e-01)	
STK3	COAD (1.20e-01)	COADREAD (1.87e-01)
	UCEC (1.40e-01)	
WWC1	STAD (2.15e-01)	STES (1.60e-01)
WWTR1	COAD (7.01e-03)	COADREAD (1.11e-02)
	PAAD (9.03e-11)	

c **FAT family expression levels in normal and HNSCC tissues**

TCGA HNSCC RNAseq, (n=564)

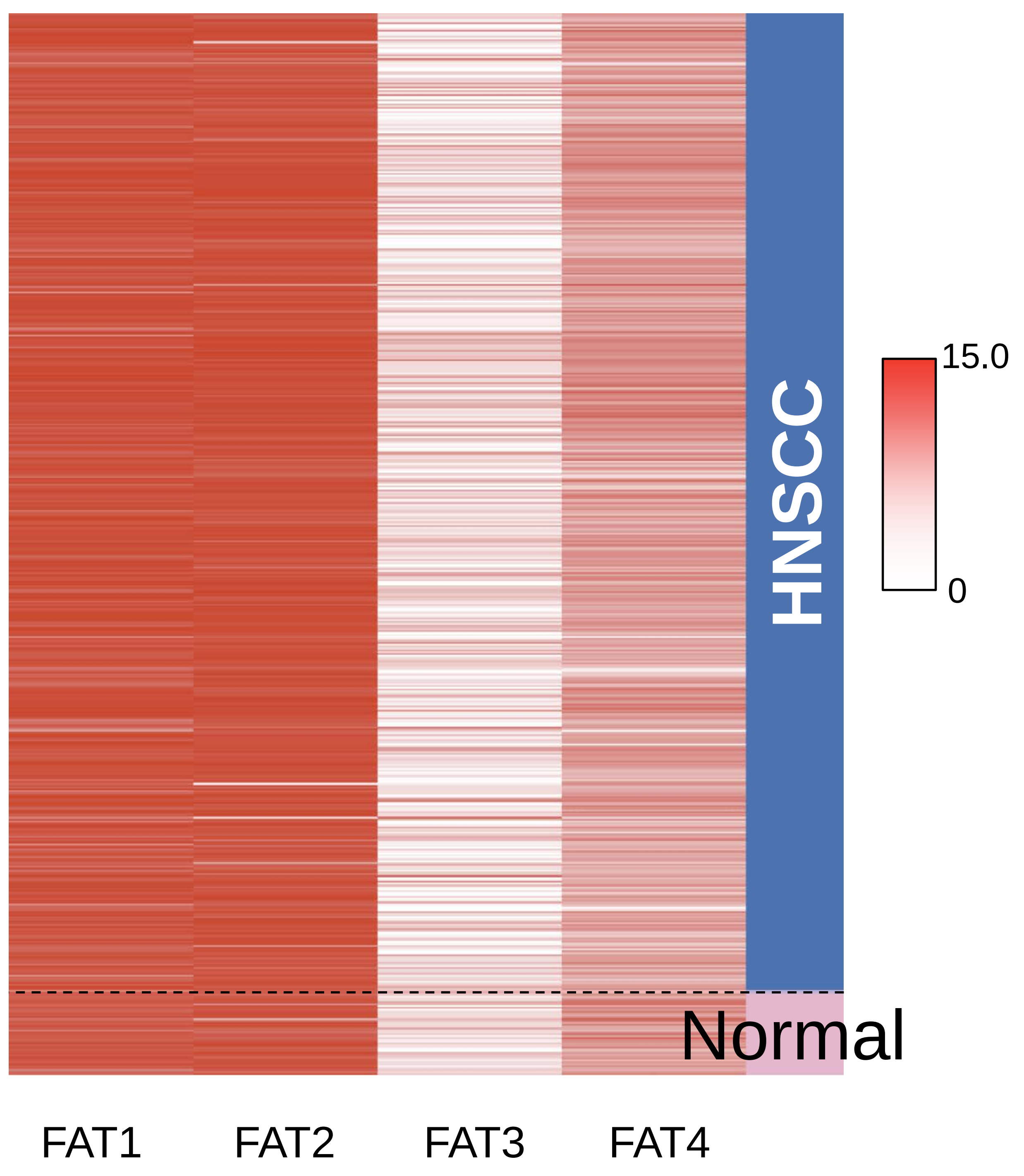

Supplementary Figure 1. Pancancer analysis of genomic alterations in candidate Hippo pathway genes. a, Pancancer analysis of candidate Hippo pathway components depicting the significance of alterations including mutations (MUTSIG) and copy number variations (CNV) type, gene copy deletion (D) or amplification (A) and significance based on GISTIC analysis. **b**, Cancer-specific mutations in candidate Hippo pathway genes and their corresponding significance (MUTSIG). C, Expression of FAT family members by RNASeq.

Supplementary Figure 2

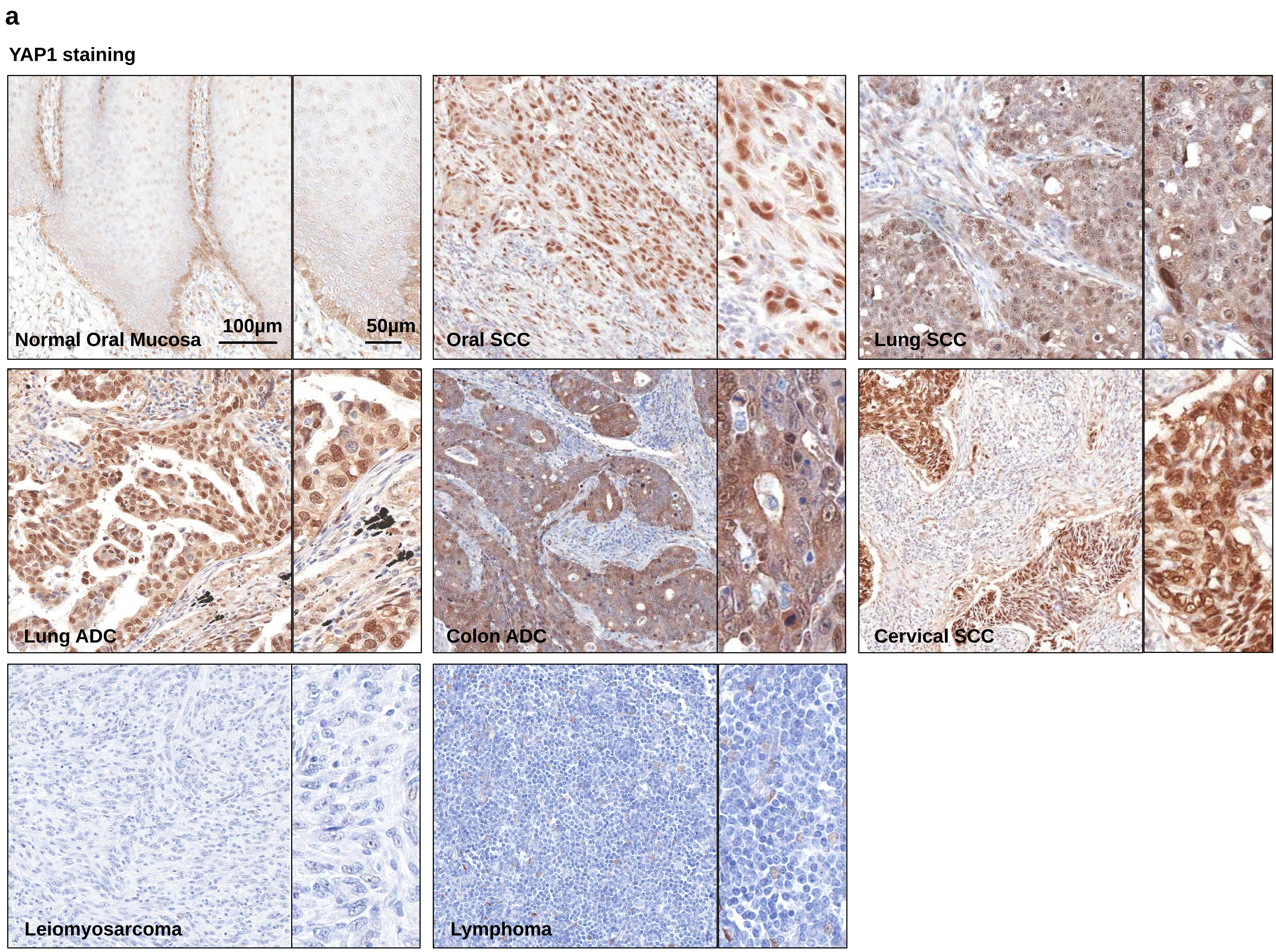

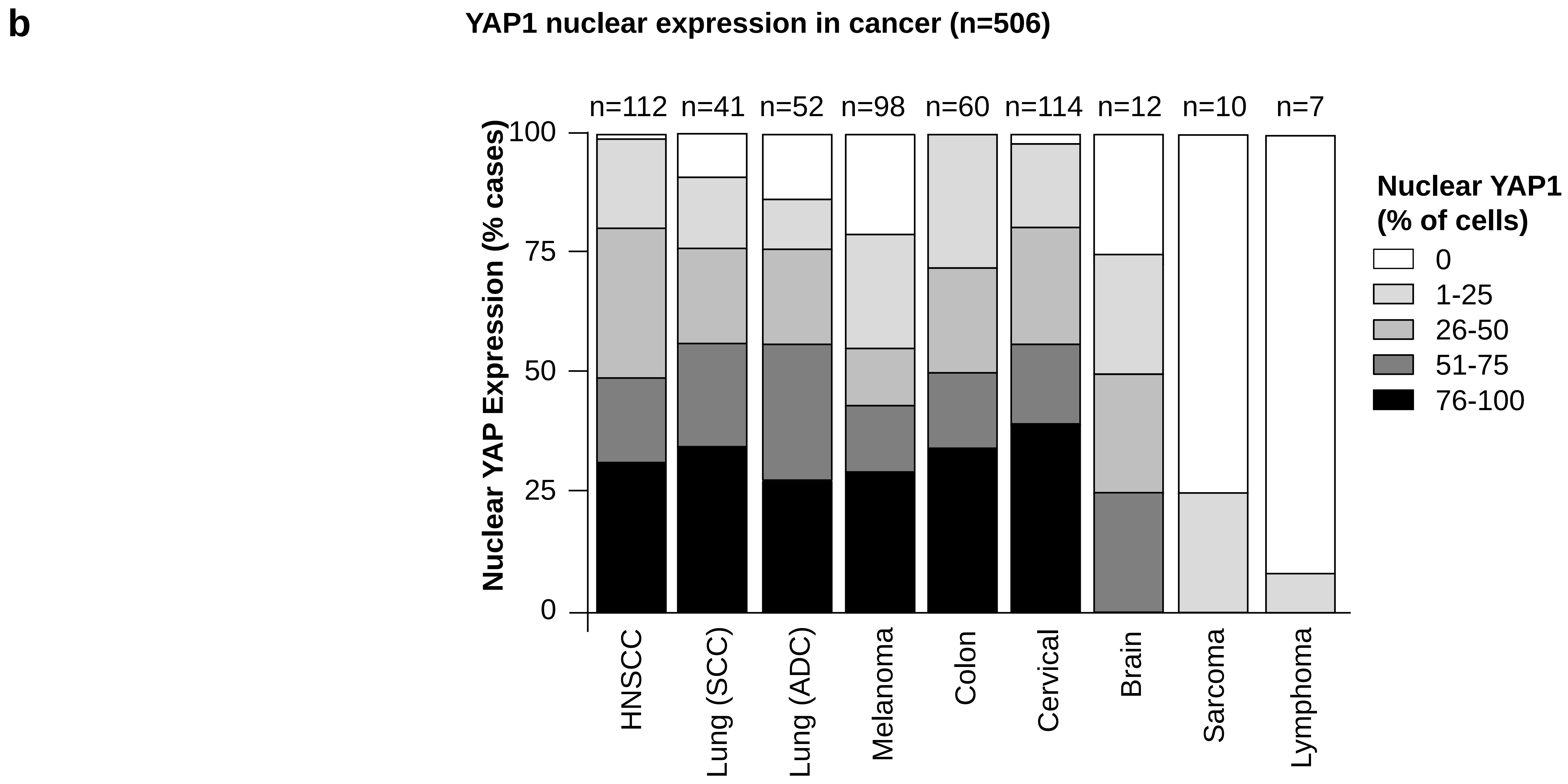

Extended data Figure 2. Multicancer analysis of YAP1 expression and localization patterns. a, Representative YAP1 stainings from multiple tissue arrays are shown for select cancer types. **b**, Quantification of nuclear-localized (active) YAP1 in the different tumor cohorts. Stacked bars represent the percentage of cases showing different proportions of nuclear localized YAP1 cells within the lesions (graded black to white colors).

Supplementary Figure 3

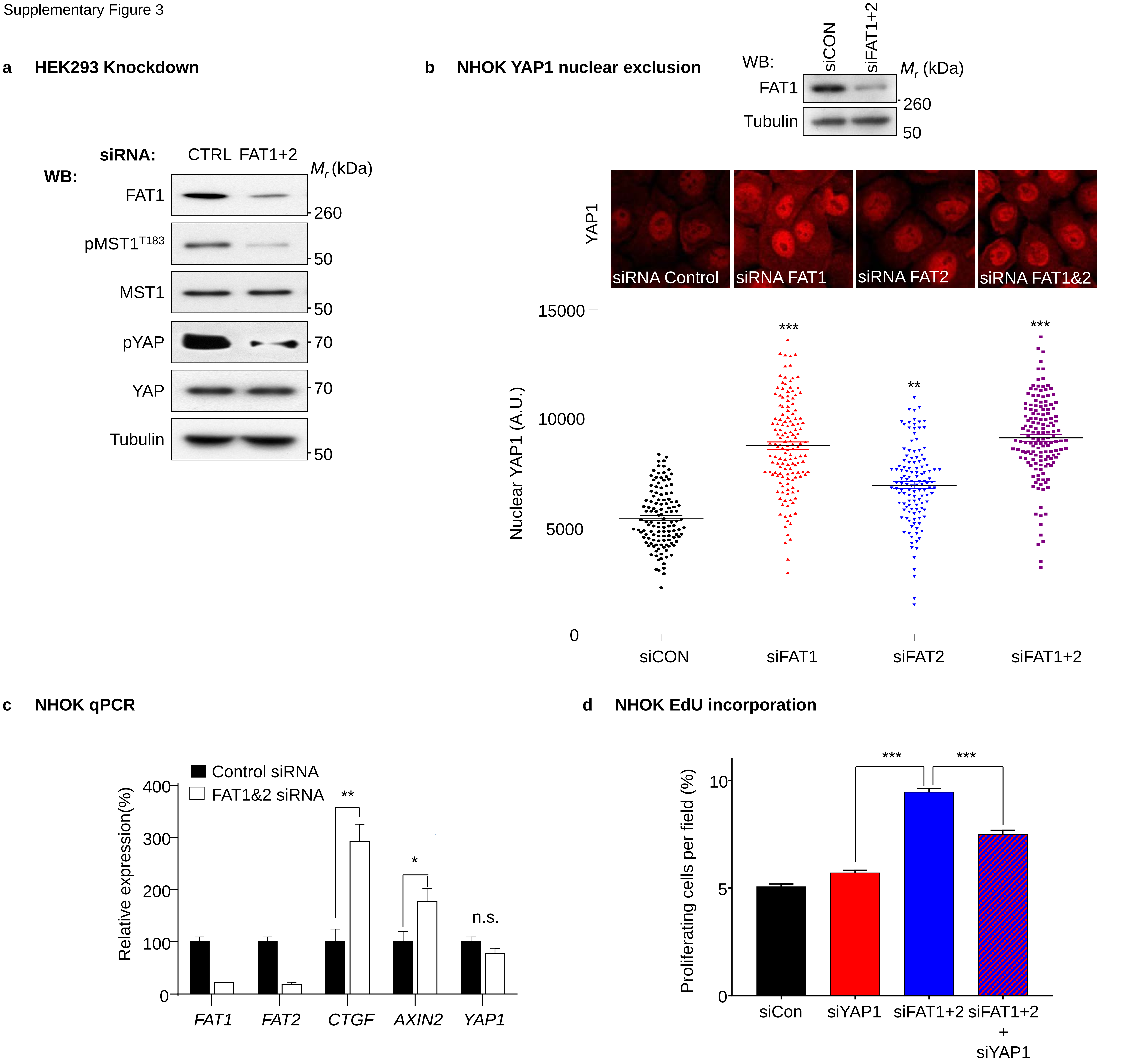

Supplementary Figure 3. Impact of knockdown of FAT1 and FAT2 in HEK293 and normal oral human oral keratinocytes (NHOKs). a, Exponentially growing HEK293 were transfected with control (CTRL) or FAT1 and FAT2 (FAT1+2) siRNAs for 48h and then lysed and analyzed by Western blot. Representative Western blots of MST1 and YAP1 are shown. **b**, NHOKs were transfected with siRNAs against control (siCON) and FAT1, FAT2 or both FAT1 and FAT2 (siFAT1+2) and cultured for 48h. Upper panel, protein lysates were prepared to confirm the knockdown of FAT1 by Western blot. Lower panel, samples treated in parallel as above were processed for immunofluorescence analysis of YAP1 localization. In red YAP1 (Alexa 546) and in blue DAPI nuclear counterstain. Right panel, Automated quantification of nuclear-localized YAP1 over a minimum of 100 cells. Black lines indicate mean nuclear YAP1 intensity ± SEM. **c**, Quantitative PCR depicting gene expression levels of the YAP1 transcriptional targets *CTFG* and the β-catenin target *AXIN2* as well as confirmation of knockdown of *FAT1* and *FAT2* in NHOK cells treated as in a. Bars represent the GAPDH-normalized mean ± SEM (N=3). **d**, NHOKs were transfected with siRNAs against control (siCON), YAP1 (siYAP1), both FAT1 and FAT2 (siFAT1+2) and YAP1 + FAT1 and FAT2 (siFAT1+2 + siYAP1) and cultured for 48h. Cell proliferation was determined by EdU incorporation (Click-it assay). The bars represent the mean ± SEM percentage of cells per field displaying nuclear incorporation of the dye as determined by automated quantification of nuclear EdU over a minimum of 100 fields. *P<0.05, **P<0.01, ***P<0.001 (One-way ANOVA).

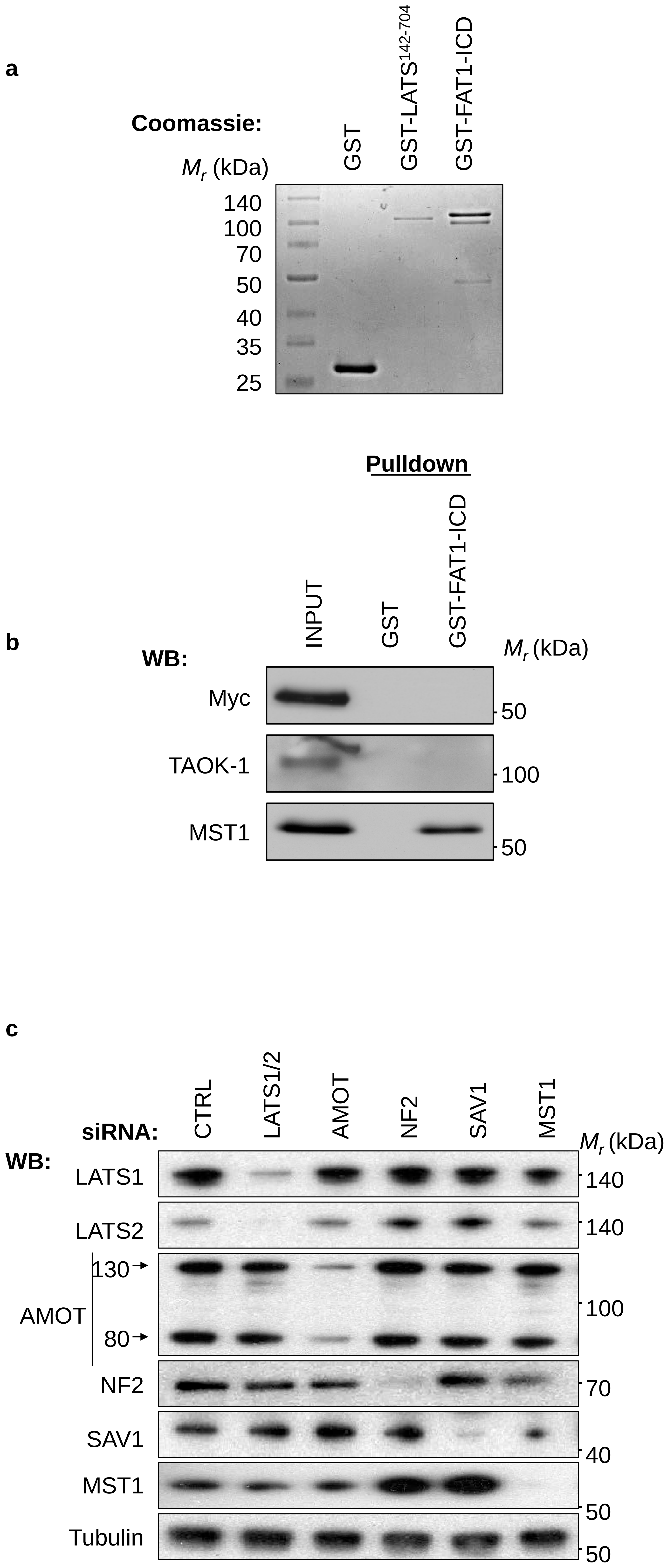

Supplementary Figure 4. Expression of GST-FAT1-ICD and GST-LATS in BL21 cells, and lack of association of TAOK1 to FAT1. a, Coomasie staining depicting the expression of all GST fusion proteins used for the pulldown experiments in Fig. 3d and 3e. **b,** Pulldown assay in HEK293 cell and analysis of the interaction with TAOK-1 by Western blot. Myc-tagged TAOK-1 was transfected in HEK293 cells and lysates were submitted to pulldown assays as indicated. **c**, siRNA-mediated knockdown on HEK293 of the different components of the Hippo signaling pathway.

a

Co-IP of Hippo signaling complexes with FAT1-TM/ICD

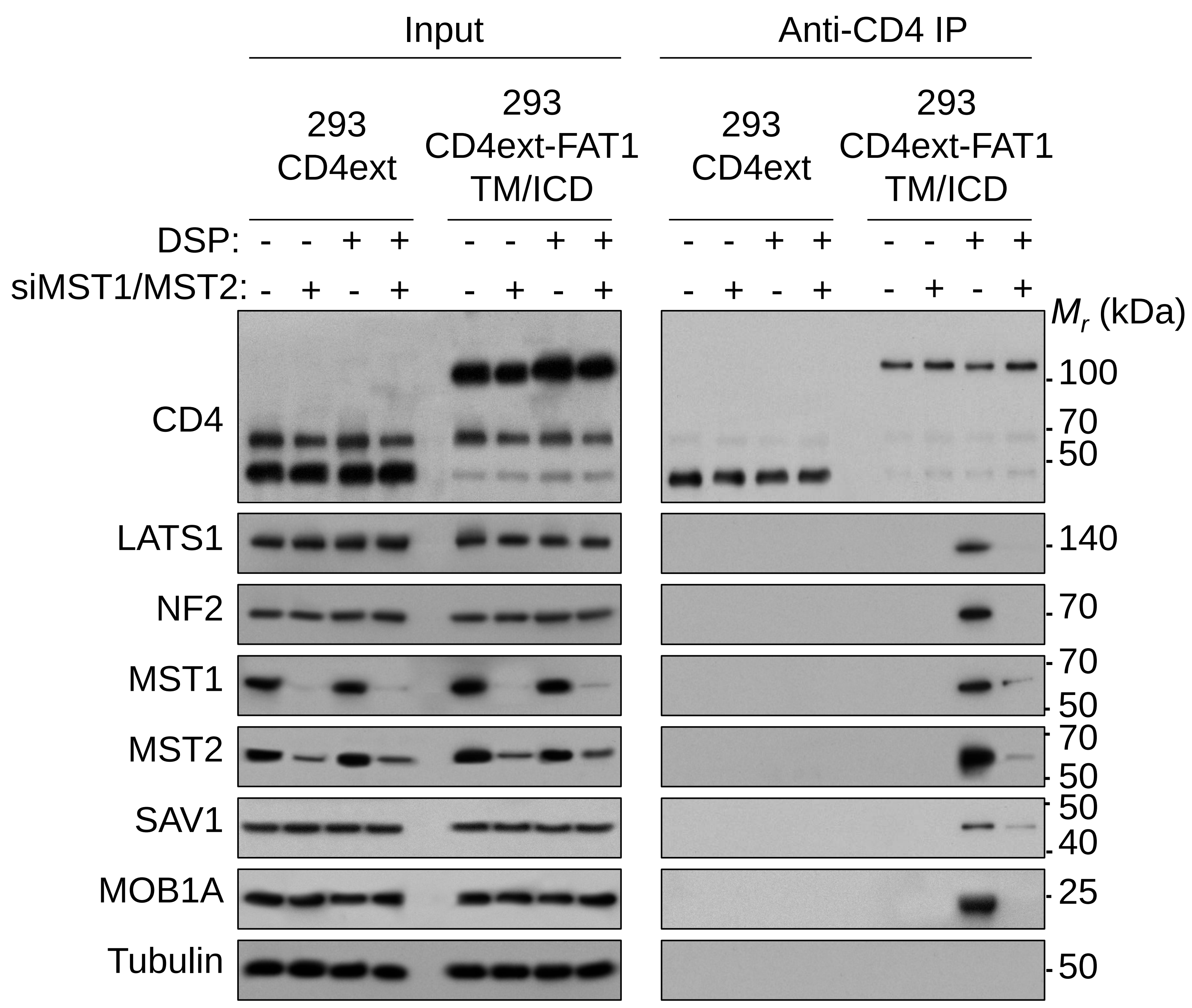

b

Co-IP with endogenous MST1

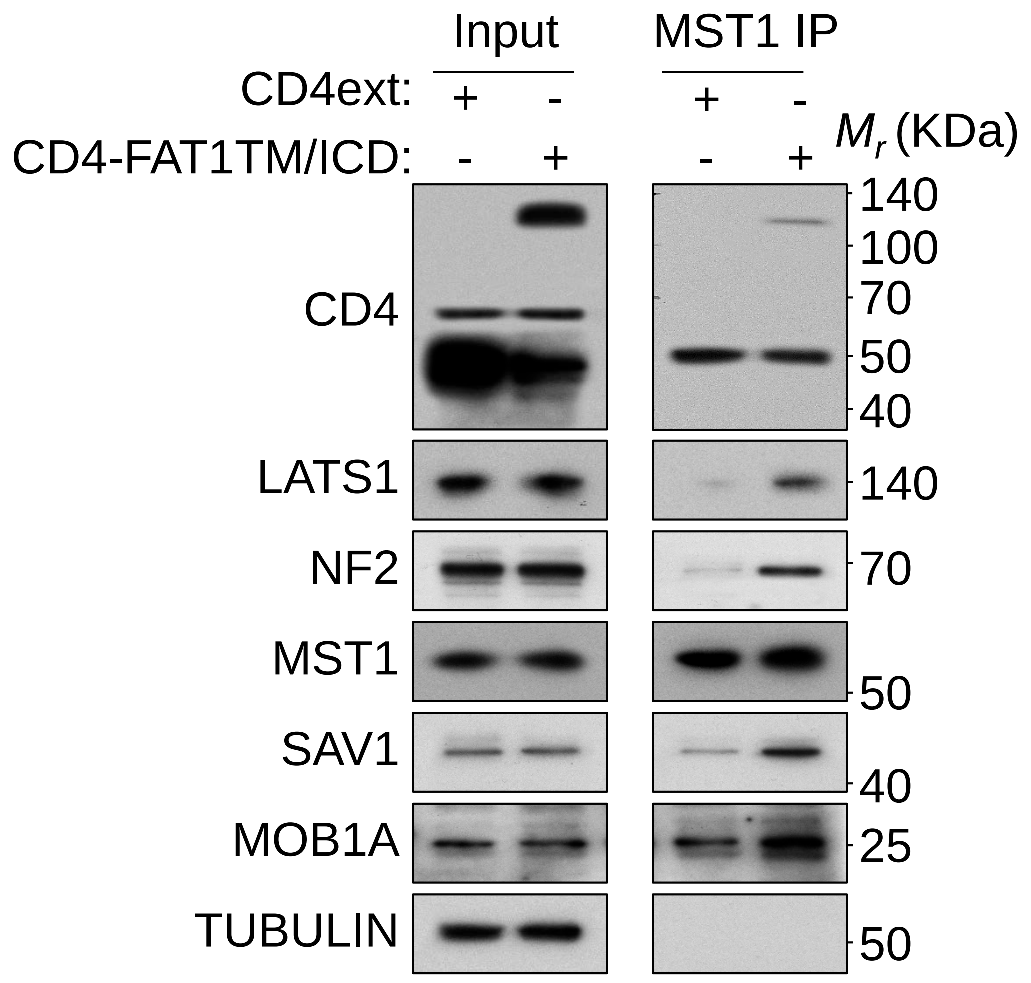

Supplementary Figure 5. Co-immune precipitation of Hippo signaling complexes with FAT1 ICD *in vivo*. a, CD4-FAT1-TM/ICD immunoprecipitation. Exponentially growing HEK293 stably expressing CD4ext or CD-FAT1-TM/ICD were transfected with MST1 and MST2 siRNAs for 48h and then treated for 2h at 4°C with DMSO (-) or the reversible crosslinker DSP (+) prior to cell lysis and immunoprecipitation with anti-CD4. Representative Western blots of the immunoprecipitated Hippo pathway members are shown. **b**, Endogenous MST1 imunoprecipitation. Exponentially growing HEK293 stably expressing CD4ext or CD-FAT1-TM/ICD were treated for 2h at 4°C with DMSO (-) or the reversible crosslinker DSP (+) prior to cell lysis and immunoprecipitation with anti-MST1. Representative Western blots of the immunoprecipitated CD4-FAT1-TM/ICD and Hippo pathway members are shown.

Supplementary Figure 6

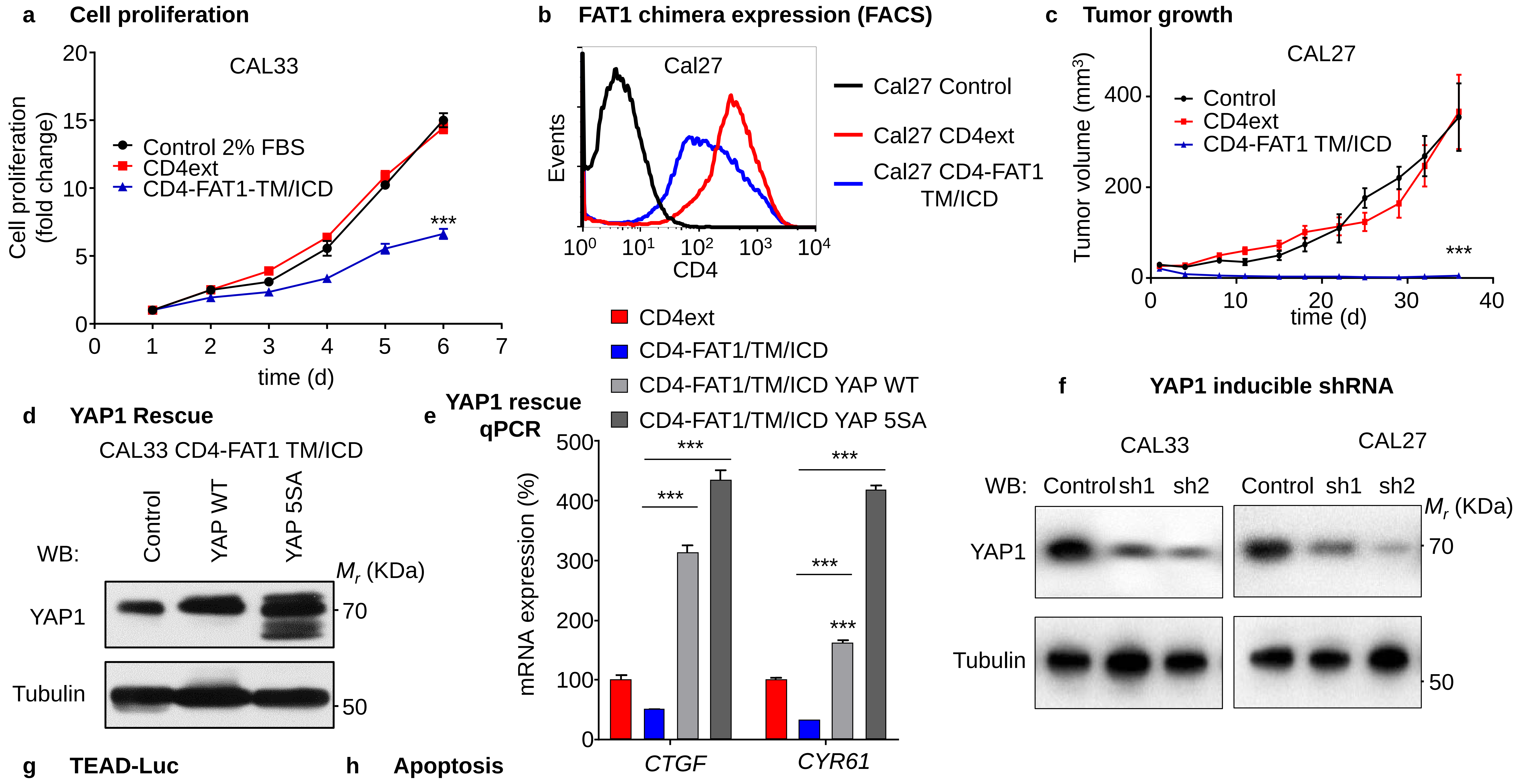

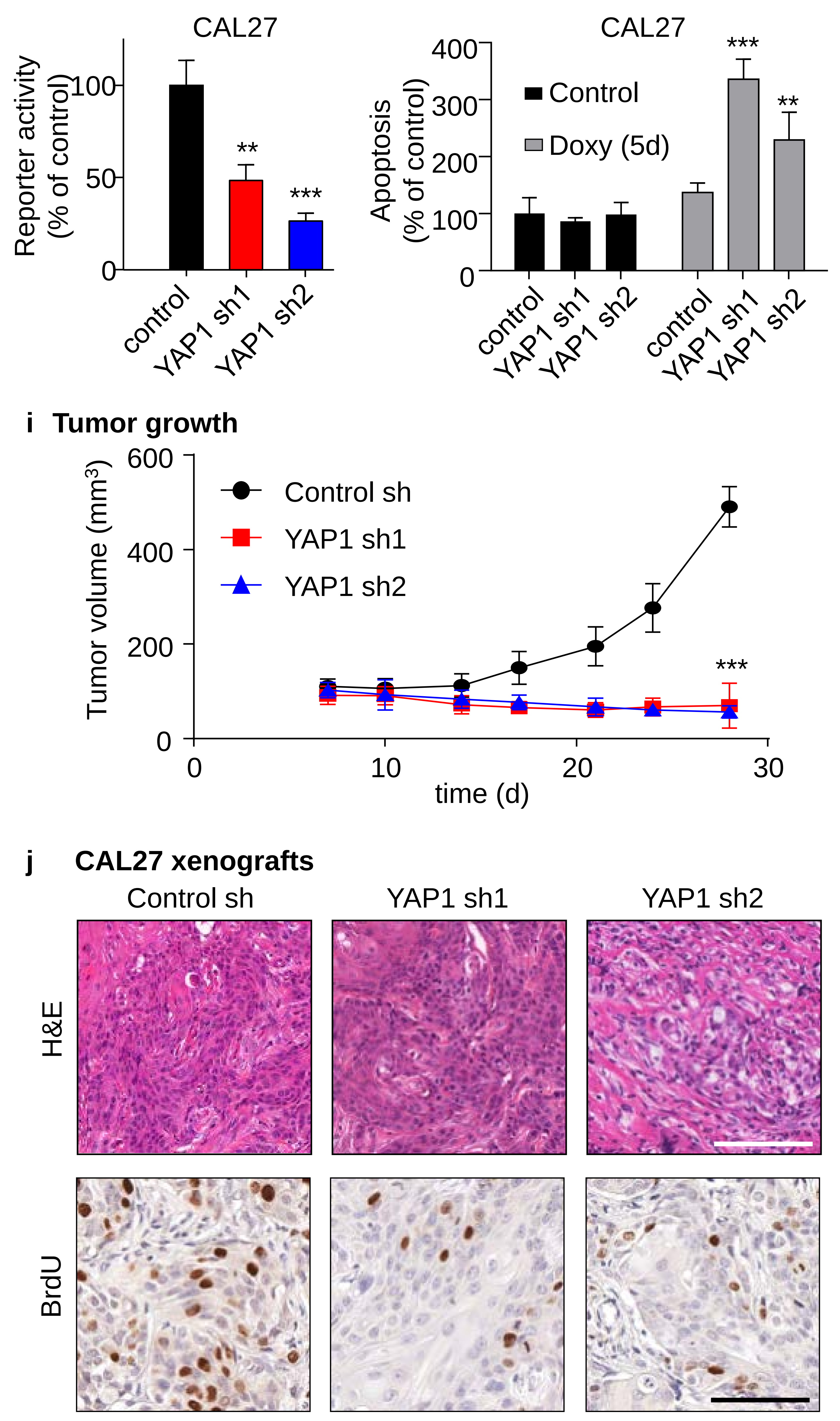

Supplementary Figure 6. Overexpression of FAT1 or downregulation of YAP1 impact cell survival and tumorigenesis. a, Cell proliferation assay on CAL33 cells stably expressing the CD4ext and CD4-FAT1-TM/ICD chimeras. Cells were cultured in the presence of 2% FBS and counted daily as indicated. Data points represent the mean fold change ± SEM (N=3) with respect to the initial number of cells. **b**, CAL27 stably transfected with the CD4ext and CD4-FAT1-TM/ICD chimeras display correct expression and localization as depicted by CD4 FACS analysis. **c**, In vivo flank xenograft assay. One million CAL27 cells were injected in the flank of *nu/nu* mice. Data points represent mean volume per group (N=10 tumors) ± SEM. **d**, CAL33 cells were stably transfected with constructs encoding YAP1 wild type (YAP1 WT) or a YAP1 version in which the S61, S109, S127, S164 and S381 residues have been mutated to Alanine and is insensitive to regulation by MST kinases and the Hippo signaling pathway (YAP1 S5A). These cells display correct expression of the constructs as depicted by a representative Western blot. **e**, Gene expression determination by quantitative PCR of YAP1 target genes in control (CD4ext), CD4-FAT1-TM/ICD expressing or YAP1 WT and YAP1 5SA overexpressing CAL33 cells VP. Bars represent normalized relative mean ± SEM gene expression (N=4). **f**, Doxycycline-dependent shRNA-mediated knockdown of YAP1 in CAL33 and CAL27 following infection and selection with inducible lentiviral YAP1 shRNA viruses. CAL33 and CAL27 cells stably expressing control and YAP1 shRNAs were stimulated with doxycycline for 5 days (1µg/ml) and then analyzed by Western blot. **g**, CAL27 stably expressing control and *YAP1* shRNAs were stimulated with doxycycline for 5 days (1µg/ml) and then transfected with a 8xTEAD-luciferase reporter. Renilla-normalized reporter activity is expressed as % of control. Bars represent mean ± SEM (N=4). **h**, Apoptosis assay by propidium iodide staining of CAL27 cell lines expressing control or *YAP1* shRNA after 5d of Doxycycline stimulation. Bars represent mean ± SEM (N=4). **i**, *In vivo* flank xenograft assay. One million cells were injected in the flank of *nu/nu* mice. Animals were fed Doxycycline food (6g/Kg) *ad libidum* 24h hours after tumor cell injection for the duration of the experiment. Data points represent mean volume per group (N=10 tumors) ± SEM. **j**, Representative immunohistochemical stainings of CAL27 tumors from panel g. $^{**}P<0.01$, $^{***}P<0.001$ (One-way ANOVA). Scale bar, white=200µm, black=100µm.

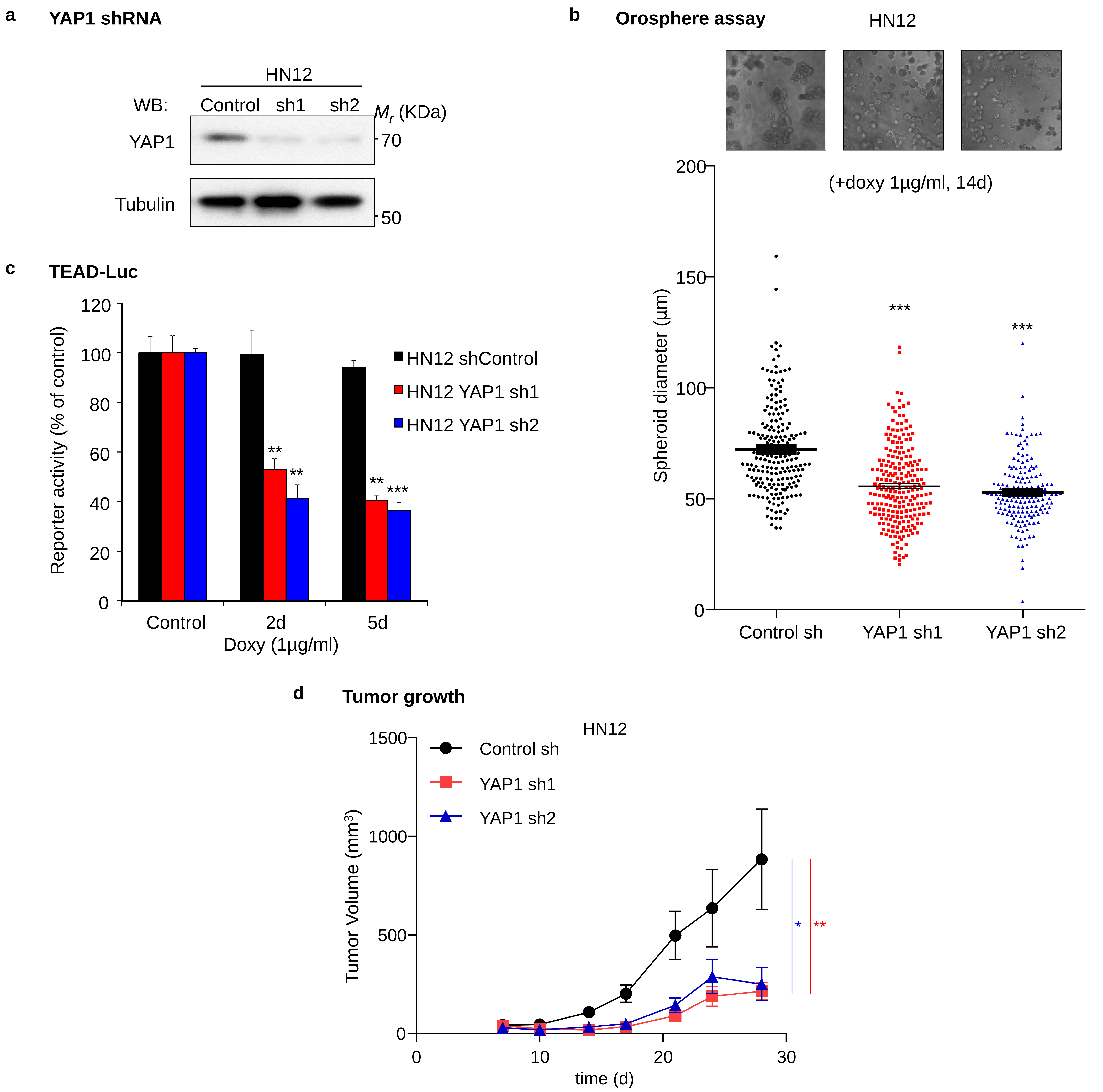

Supplementary Figure 7. YAP1 shRNA reduces HN12 proliferation and tumor growth. a, Doxycycline-dependent shRNA-mediated knockdown of *YAP1* in HN12 following infection and selection with inducible lentiviral *YAP1* shRNA viruses. HN12 stably expressing control and YAP1 shRNAs were stimulated with doxycycline for 5 days (1µg/ml) and then analyzed by Western blot. **b**, Spheroid formation assay of stable HN12 shRNA control and *YAP1* shRNA cell lines. Representative pictures are shown on top and diameter quantifications (>200 colonies per group) are shown below. Black lines represent mean ± SEM. **c**, HN12 stably expressing control and *YAP1* shRNAs were stimulated with doxycycline (1µg/ml) for 5d and then transfected with a 8xTEAD-luciferase reporter. Renilla-normalized reporter activity is expressed as % of control. Bars represent mean ± SEM (N=4). **d**, *In vivo* flank xenograft assay. One million cells were injected in the flank of *nu/nu* mice. Animals were fed Doxycycline food (6g/Kg) *ad libidum* 24h hours after tumor cell injection for the duration of the experiment. Data points represent mean volume per group (N=10 tumors) ± SEM. *P<0.05, **P<0.01, ***P<0.001 (One-way ANOVA).

Supplementary Figure 8

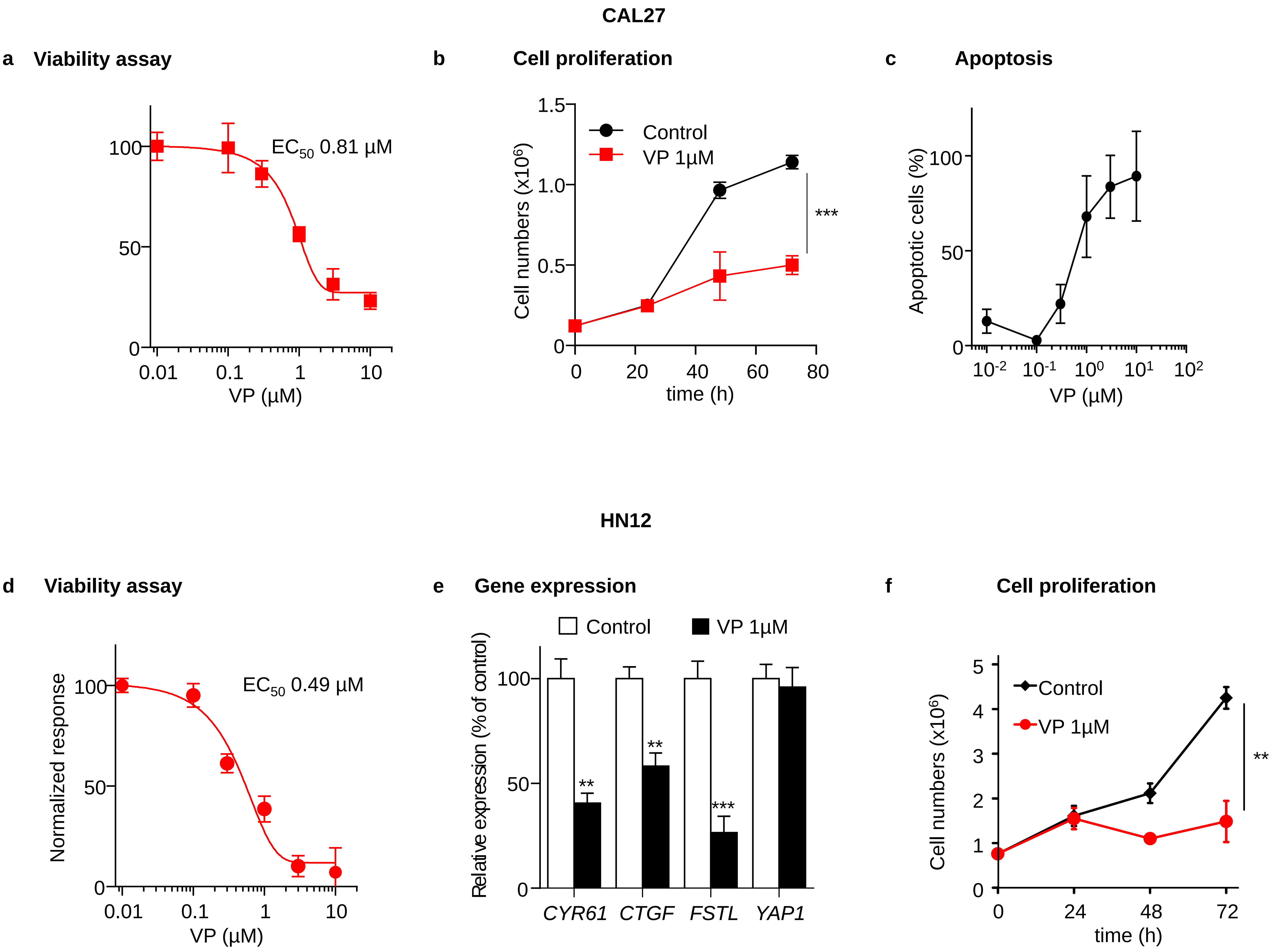

Supplementary Figure 8. Verteporfin decreases viability, proliferation and YAP1-dependent gene expression in Cal 27 and HN12 cells. a, Dose-response experiment for cell viability as determined by the AlamarBlue assay in CAL27 cell lines subjected to 48h treatments with VP. Data points represent mean ± SEM (N=8). **b,** Proliferation assay by cell counting of CAL27 cells exposed to vehicle (Control) or 1µM VP for the times indicated. Data points represent mean ± SEM (N=4). **d,** Apoptosis assay by propidium iodide staining. Dose-dependent VP-induced apoptosis at 48h in CAL27 HNSCC cell. Data points represent mean ± SEM (N=3). **d,** Dose-response experiment for cell viability as determined by the AlamarBlue assay in HN12 cell lines subjected to 48h treatments with VP. Data points represent mean ± SEM (N=8). **e**, Gene expression determination by quantitative PCR of YAP1 target genes after 18h treatment with 1µM VP. Bars represent mean ± SEM (N=4). **f**, Proliferation assay by cell counting of HN12 cells exposed to 1µM VP for the times indicated in the figure. Data points represent mean ± SEM (N=4). ***P*<0.01, ****P*<0.001 (One-way ANOVA).

Supplementary Figure 9 | Uncropped Western blots from Figure 2

Figure 2b

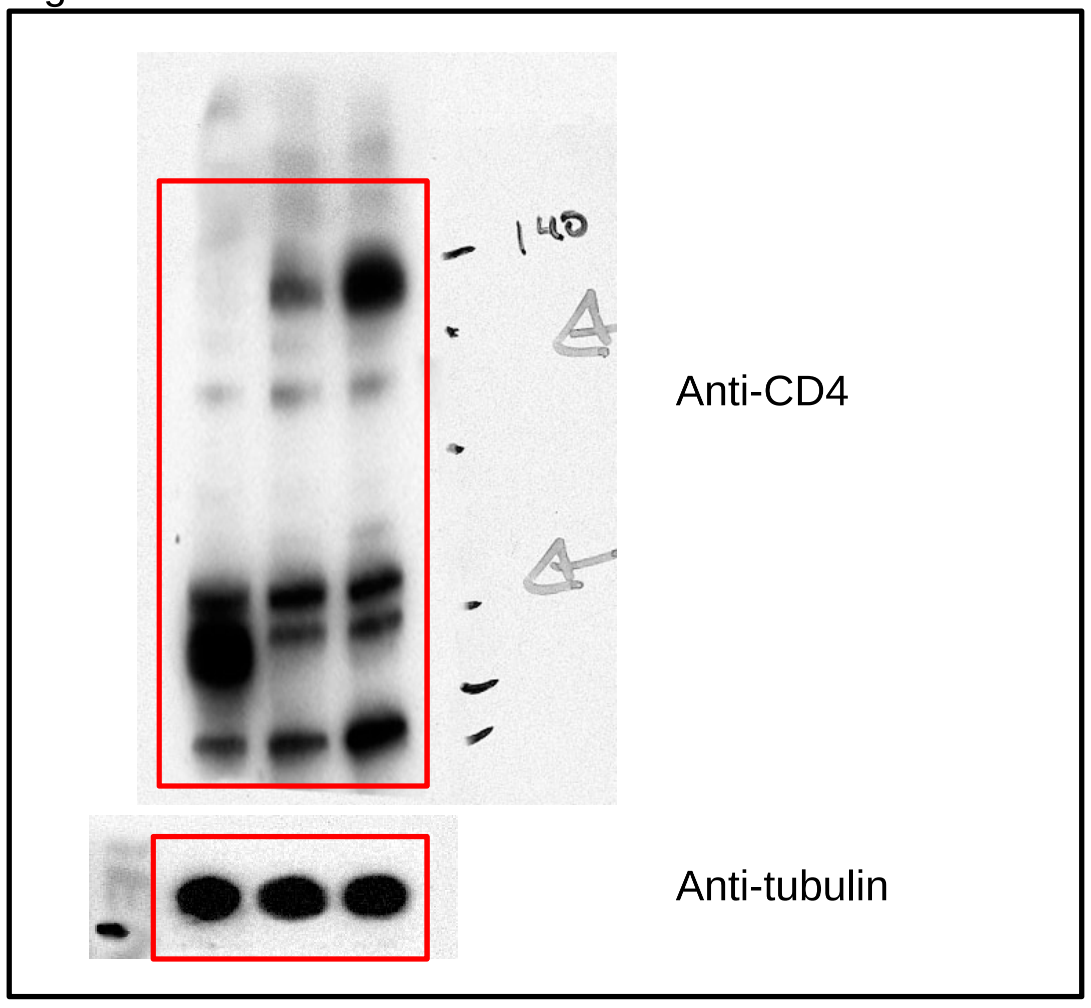

Supplementary Figure 10 | Uncropped Western blots from Figure 3

Figure 3a

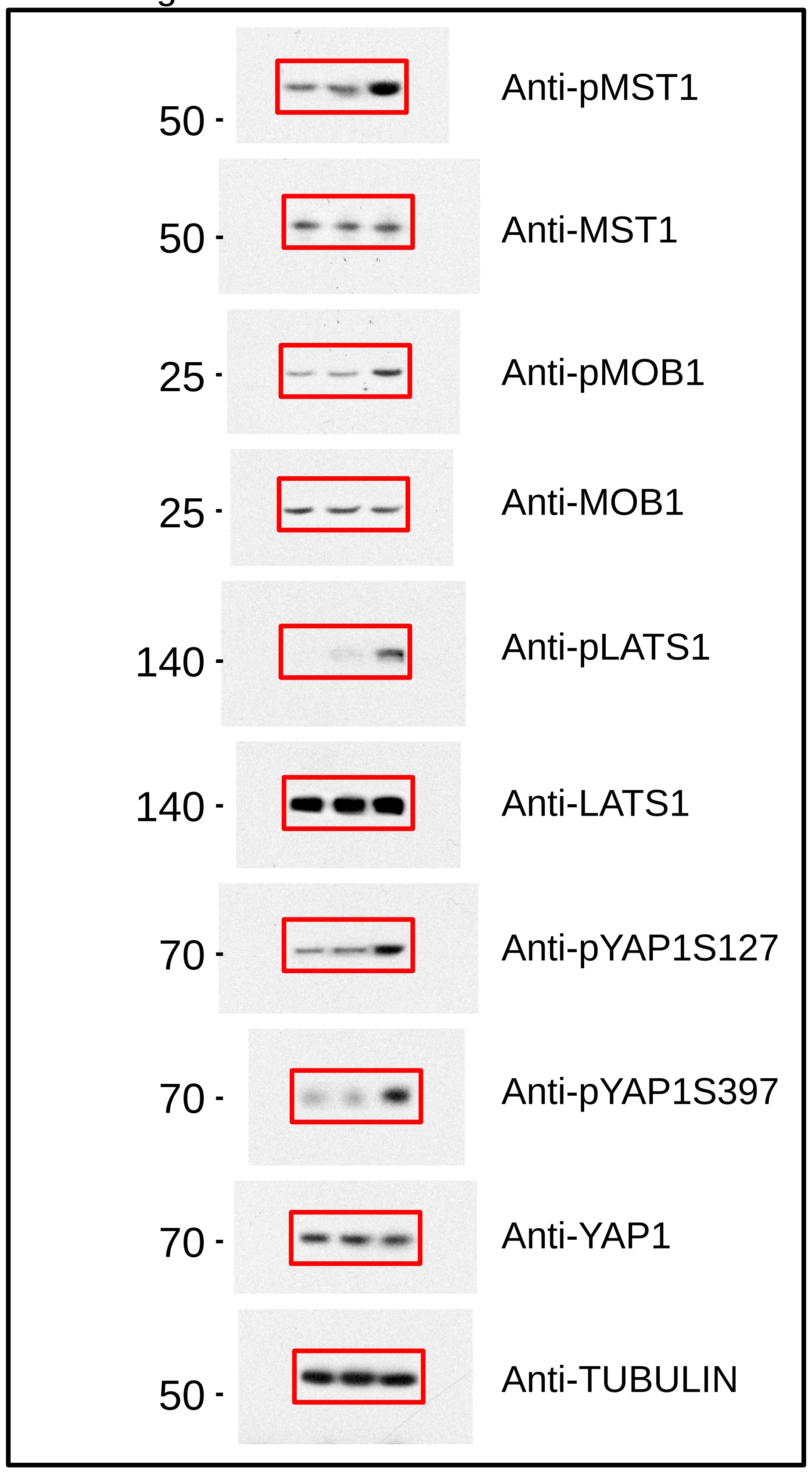

Figure 3b

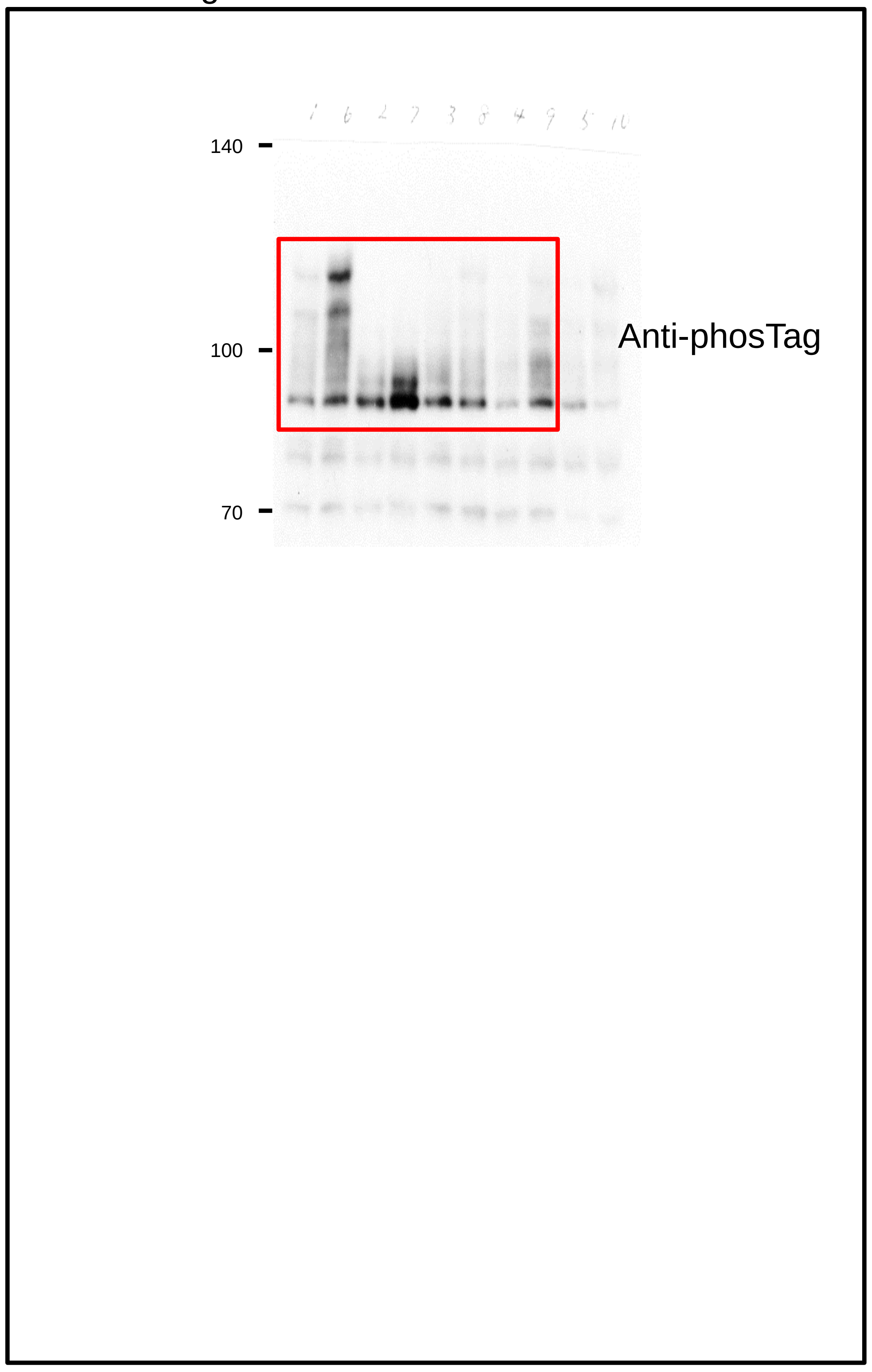

Figure 3c

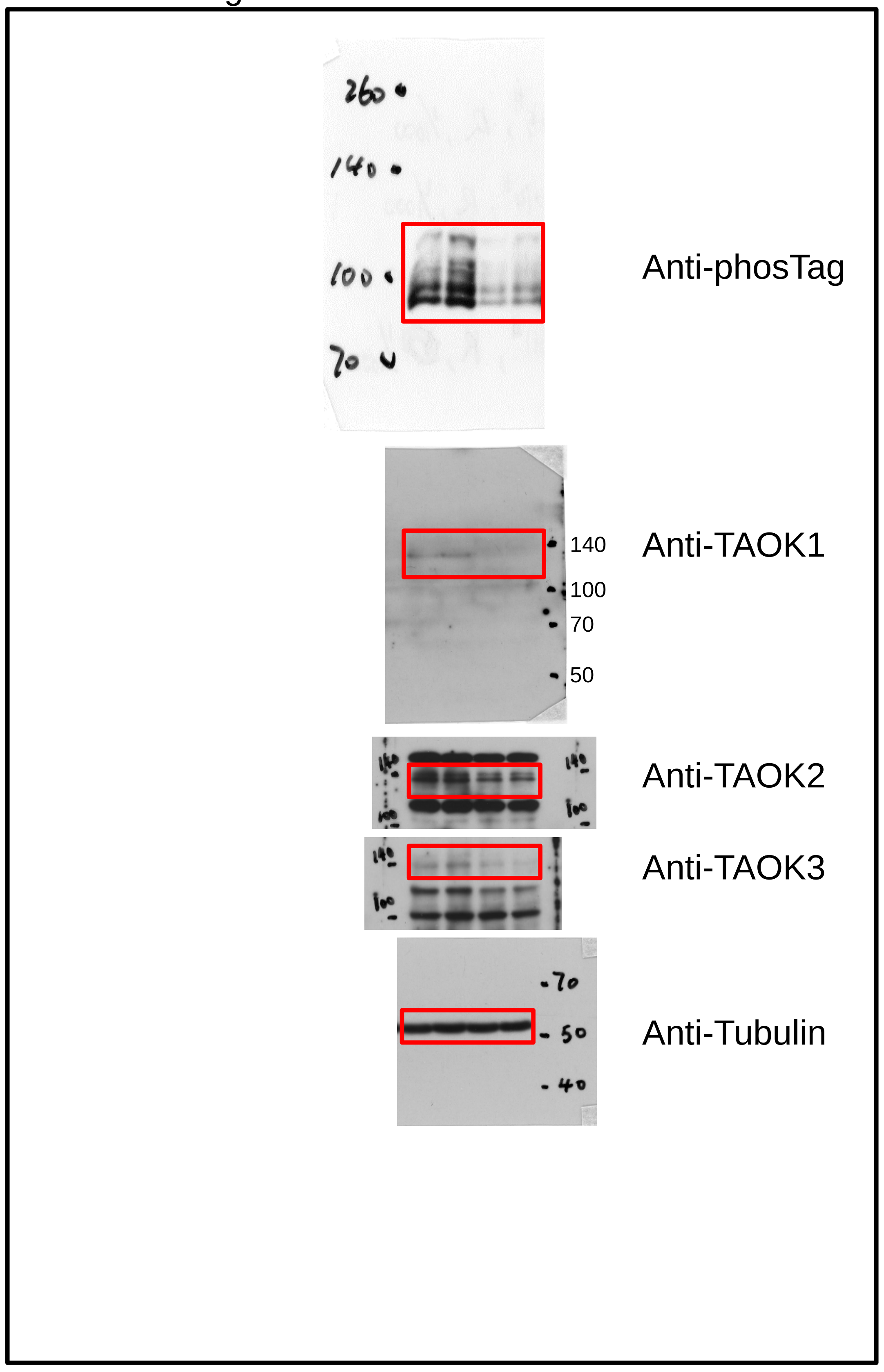

Figure 3d

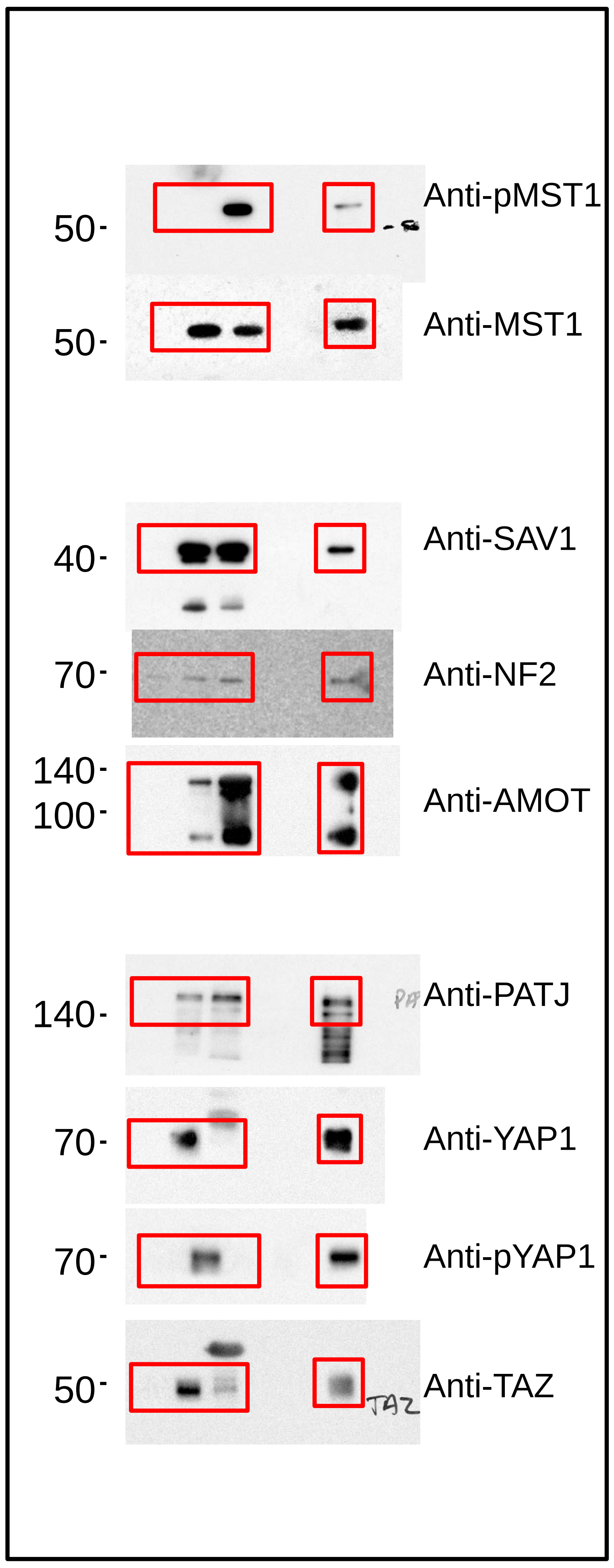

Figure 3f

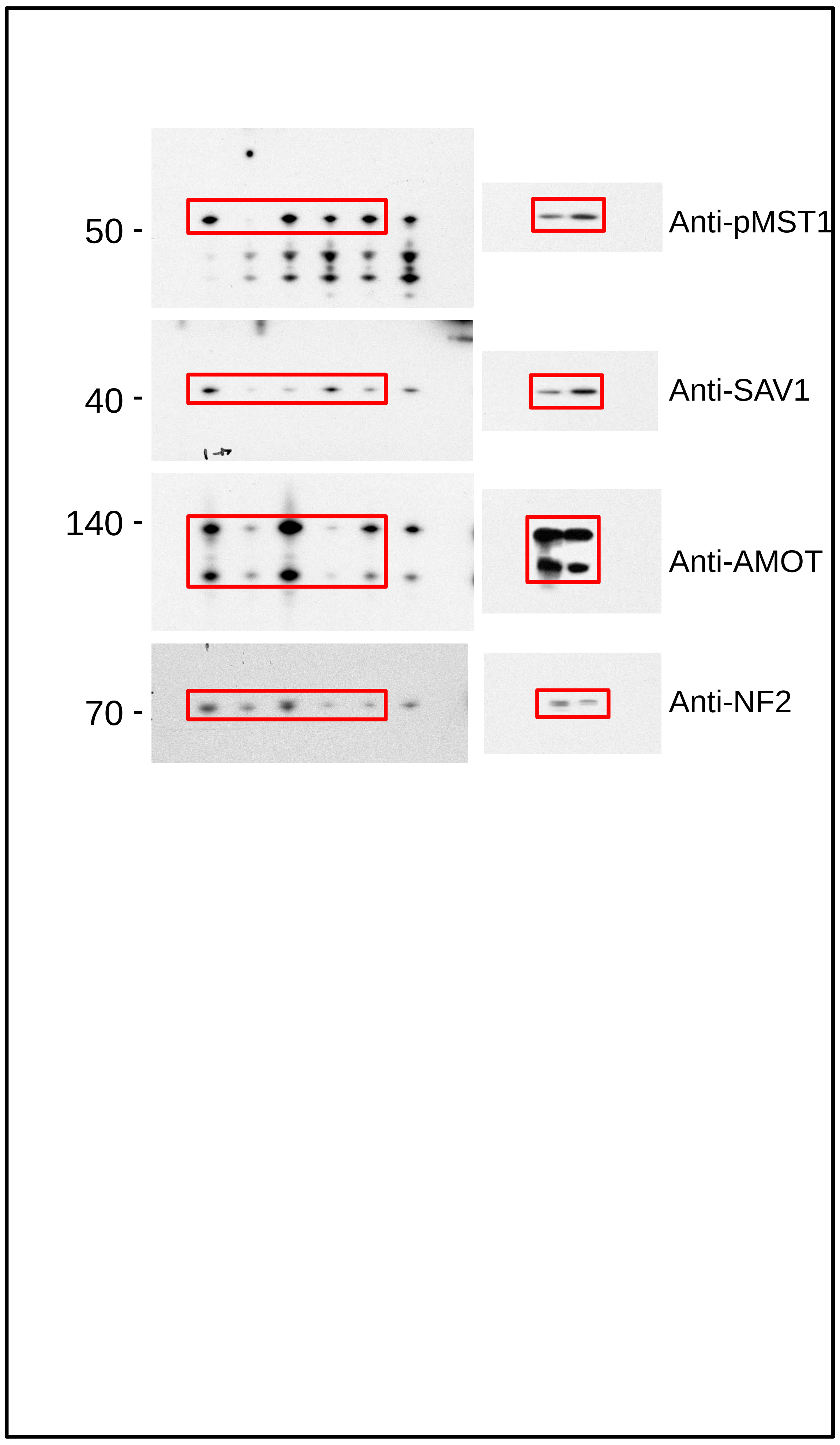

Figure 3g

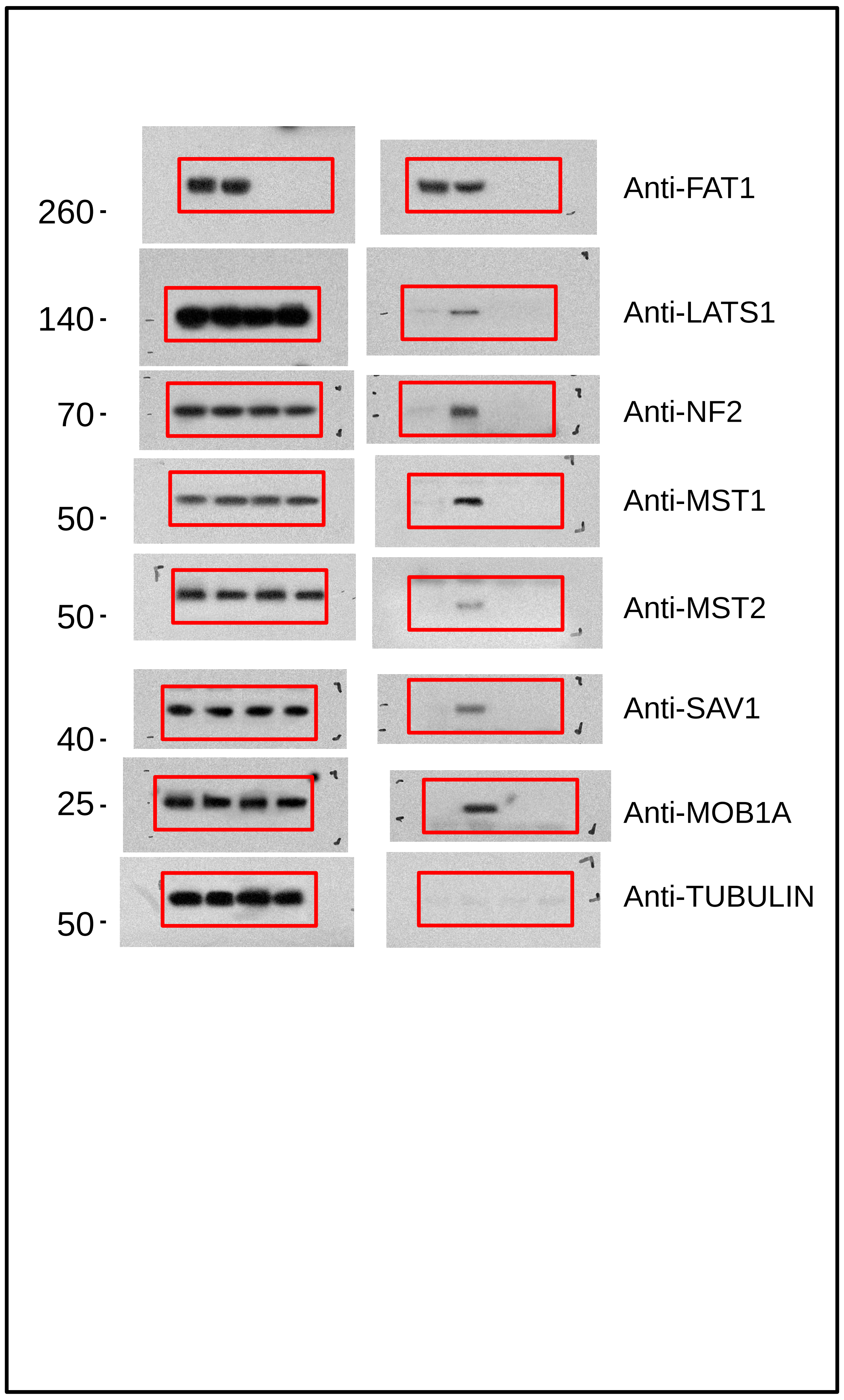

Supplementary Figure 11 | Uncropped Western blots from Figure 4

Figure 4a

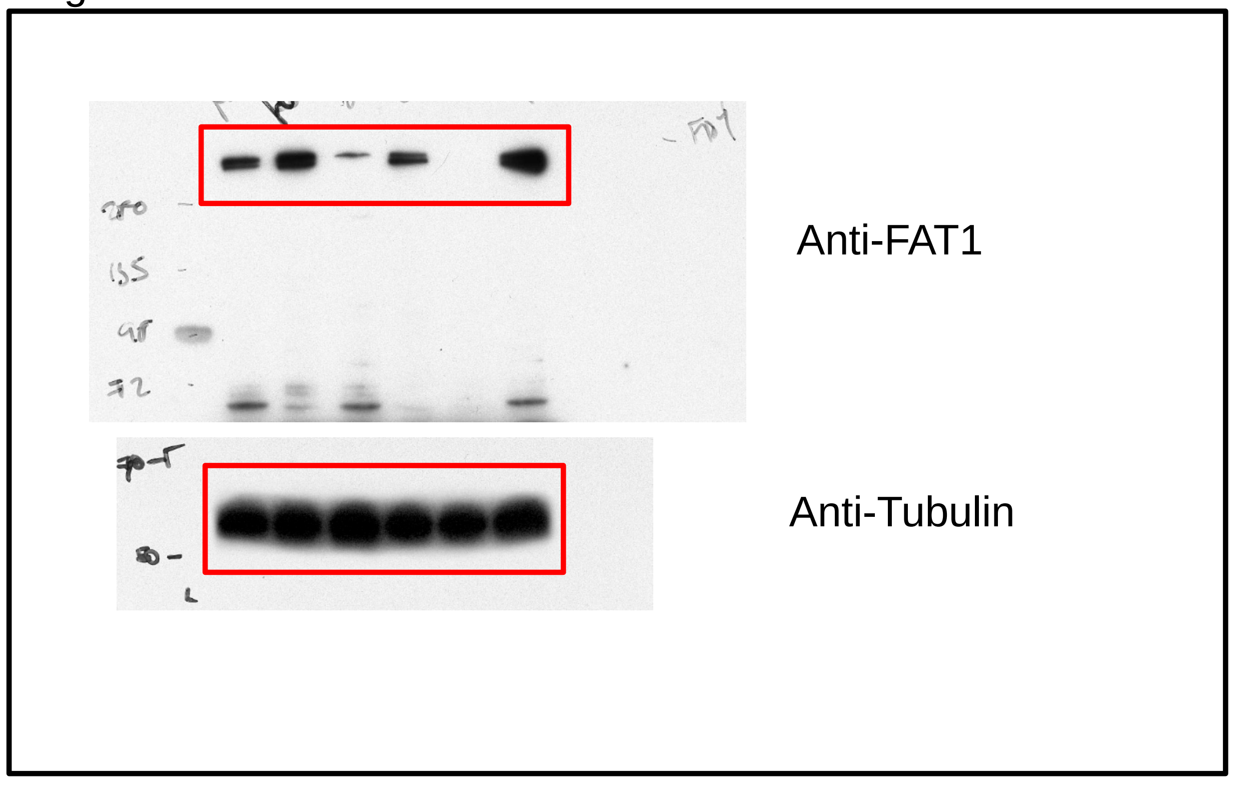

Supplement: Supplementary file 1 — Supplementary Information [file 41467_2018_4590_MOESM1_ESM.pdf]
