# Supplementary material for: Assembly and activation of the Hippo signalome by FAT1 tumor suppressor
Source: Nat Commun. 2018 Jul 9;9:2372. doi: 10.1038/s41467-018-04590-1 (PMC6037762; doi:10.1038/s41467-018-04590-1)
Supplement: Supplementary file 3 — Description of Additional Supplementary Files [file 41467_2018_4590_MOESM3_ESM.pdf]

Description of Additional Supplementary Files

File Name: Supplementary Data 1
Description: Differentially expressed genes in FAT normal versus FAT mutant HNSCC samples from TCGA.

File Name: Supplementary Data 2
Description: Results from Transfac and Jaspar PWMs Enrichr analysis.

File Name: Supplementary Data 3
Description: Sequences of oligonucleotides used.

File Name: Supplementary Data 4
Description: Cancer abbreviations and number of cases included in the Hippo members and FAT1 analysis in Figure 1.
