# Supplementary material for: Assembly and activation of the Hippo signalome by FAT1 tumor suppressor
Source: Nat Commun. 2018 Jul 9;9:2372. doi: 10.1038/s41467-018-04590-1 (PMC6037762; doi:10.1038/s41467-018-04590-1)
Supplement: Supplementary file 2 — Peer Review File [file 41467_2018_4590_MOESM2_ESM.pdf]

Editorial Note: this manuscript has been previously reviewed at another journal that is not operating a transparent peer review scheme. This document only contains reviewer comments and rebuttal letters for versions considered at *Nature Communications*.

REVIEWERS' COMMENTS:

Reviewer #1 (Remarks to the Author):

I think the manuscript is once again much improved. I have reviewed the manuscript, response to reviewers, including my prior comments and the other reviewers. I have reviewed the supplemental data. I have no further concerns and I am enthusiastic about this manuscript and the novel findings being reported.

Reviewer #3 (Remarks to the Author):

The authors have done a significant amount of work in response the the reviewer comments and have addressed my previous comments in a satisfactory manner. The manuscript is well written and their conclusions are supported by their data. Their findings are relevant and of interest to the field and the data of high quality. I support the publication of this manuscript and thank toe authors for their additional work.
